# Diversity of the Bacterial Microbiota of *Anopheles* Mosquitoes from Binh Phuoc Province, Vietnam

**DOI:** 10.3389/fmicb.2016.02095

**Published:** 2016-12-23

**Authors:** Chung T. Ngo, Sara Romano-Bertrand, Sylvie Manguin, Estelle Jumas-Bilak

**Affiliations:** ^1^Institut de Recherche pour le Développement France, UMR-MD3, Faculté de PharmacieMontpellier, France; ^2^National Institute of Veterinary ResearchHanoi, Vietnam; ^3^UMR 5569 Hydrosciences, Equipe Pathogènes Hydriques, Santé et Environnements, Faculté de Pharmacie, Université de MontpellierMontpellier, France; ^4^Département d’Hygiène Hospitalière, Centre Hospitalier Universitaire de MontpellierMontpellier, France

**Keywords:** *Anopheles* mosquitoes, abdomen bacterial microbiota, 16S rRNA PCR – TTGE, malaria, Binh Phuoc, Vietnam

## Abstract

The naturally acquired microbiota of *Anopheles* can influence vector’s susceptibility to *Plasmodium* and its capacity to transmit them. Microbiota modification is a new challenge to limit disease transmission but it still needs advanced knowledges on bacterial community in *Anopheles*, especially in wild and infected specimens from diverse origin and species. Bacterial culture and 16S rRNA gene-PCR associated to Temporal Temperature Gradient Electrophoresis (TTGE) were applied to explore the bacterial diversity in the abdomen of 100 wild specimens (eight *Anopheles* species) collected in the Binh Phuoc Province, Vietnam. Culture and PCR-TTGE were complementary. The bacterial richness of the mosquito collection encompassed 105 genera belonging to seven phyla, mostly *Proteobacteria*, *Firmicutes*, and *Actinobacteria*. *Staphylococcus*, *Clostridium*, and *Bacillus* in *Firmicutes* were the most prevalent genera. However, *Proteobacteria* represented by 57 genera was the most diversified phylum in *Anopheles* microbiota. The high overall of *Anopheles*-associated bacteria is confirmed with, to our knowledge, 51 genera described for the first time in *Anopheles* microbiota. However, the diversity per specimen was low with average diversity index and the average Shannon–Wiener score (H) of 4.843 and 5.569, respectively. The most represented bacterial genera were present in <30% of the specimens. Consequently, the core microbiota share by *Anopheles* from Binh Phuoc was very narrow, suggesting that *Anopheles* microbiota was greatly influenced by local environments. The repertory of bacterial genera in two specimens of *An. dirus* and *An. pampanai* naturally infected by *Plasmodium vivax* was also described as preliminary results. Finally, this study completed the repertory of bacteria associated to wild *Anopheles*. *Anopheles* associated-bacteria appeared specimen-dependent rather than mosquitoe species- or group-dependent. Their origin and the existence of *Anopheles*-specific bacterial taxa are discussed.

## Introduction

Despite some success in controlling malaria, this disease continues to be a major health burden in many countries around the world with 438,000 deaths reported in 2015, particularly in sub-Saharan Africa recording 395,000 deaths (90%), but also in forested regions of Southeast Asia with 32,000 deaths (7%; WHO, 2015). Until now, vector control has been one of the key elements for controlling this disease based on the use of insecticides, such as DDT, pyrethroids, organophosphates, carbamates (Verhaeghen et al., 2009; Kabula et al., 2013). However, this strategy of insecticide use has many serious side-effects, in particular on human health and the environment (Dabrowski and Balderacchi, 2013; Singleton et al., 2013; Dabrowski et al., 2014), having also a direct impact on the increase of *Anopheles* resistance to insecticides which has now been reported in 64 countries (WHO, 2012; Chang et al., 2013; Kabula et al., 2013; Tangena et al., 2013). Therefore, new and innovative approaches to control *Anopheles* vectors for reducing malaria transmission on a more durable and safer manner for human health are, hence, needed.

In Vietnam, malaria remains the most important vector-borne parasitic disease with a strong prevalence in forested regions, in particular along the international borders with Cambodia, where Binh Phuoc Province is located. This province, situated in central-south Vietnam, is considered as having the highest malarial transmission of all (Abe et al., 2009; Tran et al., 2012). The local malaria situation reported both resistance of *Plasmodium* to anti-plasmodial drugs and *Anopheles* mosquitoes to the insecticide used in this province (Huong et al., 2001; Nguyen et al., 2003; Thanh et al., 2010; Verhaeghen et al., 2010; Tran et al., 2012). However, there is a lack of information on the distribution and vector competence of the local *Anopheles* vectors.

The impact of the microbiota on mosquito infection and more specifically on *Anopheles* resistance to malaria pathogens is showing great potential toward reducing the mosquito vector competence and blocking transmission of several infectious diseases (Dong et al., 2006, 2009, 2011; Blumberg et al., 2013). The influence of the *Anopheles* microbiota on its vectorial competence to transmit pathogens is a growing field of investigation as demonstrated by the recent increase in studies, to cite a few (Favia et al., 2007; Briones et al., 2008; Dong et al., 2009; Rodrigues et al., 2010; Cirimotich et al., 2011; Blumberg et al., 2013; Gendrin and Christophides, 2013; Dennison et al., 2014; Buck et al., 2016). A previous report on the biodiversity of the abdomen microbiota of 100 wild *Anopheles* specimens (five species) from Dak Nong Province showed a high taxonomic diversity, including species reported as implicated in the mosquito resistance to *Plasmodium* infection (Ngo et al., 2015). By the same approach, we present here the bacterial diversity detected in 100 *Anopheles* specimens of eight species collected from Binh Phuoc, a Province characterized by high malaria endemicity. The *Anopheles* studied herein included two specimens naturally infected by *Plasmodium vivax*.

## Materials and Methods

### *Anopheles* Samples: Collection and Identification

One hundred *Anopheles* (out of 486 specimens collected from 4 sites located in Bu Gia Map District, Binh Phuoc Province in central-southern Vietnam (Ngo et al., 2014), which belonged to eight *Anopheles* species, including *Anopheles dirus*, *An. jeyporiensis*, *An. maculatus*, *An. minimus*, *An. pampanai*, *An. rampae*, *An. sawadwongporni*, and *An. scanloni*, were randomly chosen for the present study. These *Anopheles* specimens were collected between November and December, 2011 (rainy-beginning of the dry season) during 11 consecutive nights (17:00–20:00 h using methods, such as light trap capture and human landing catches indoor and outdoor (Ngo et al., 2014).

*Anopheles* mosquito identification was morphologically done in the field by sorting out each taxon. Specimens that belonged to the Dirus Complex or the Funestus and Maculatus Groups were individually identified to species level using the appropriate PCR-based assay as described by Walton et al. (1999, 2007) and Garros et al. (2005). Each individual was split into two pieces stored at -80°C until use: (1) head-thorax for species identification and detection of *Plasmodium* by molecular methods (Ngo et al., 2014); and (2) abdomen for bacterial analysis. One hundred abdomens of wild-caught *Anopheles* females were used for the subsequent bacterial study by both methods, culture and DNA fingerprint, as described below.

### Bacterial Culture, DNA Extraction, and Strains Identification

*Anopheles* abdomens were surface rinsed twice in sterilized DNA-free water, and each abdomen was thoroughly disrupted using a sterilized tissue crusher device in 150 μl of sterile DNA-free water. Then, 10 μl of this suspension was spread on each prepared culture medium plate: blood sheep agar, R2A and *Acetobacter* agar. The detailed protocol followed the one published by Ngo et al. (2015). In order to identify strains, 16S rRNA gene was amplified using the Taq DNA polymerase (Go Taq Promega) and the universal primer pair (27f and 1492r) as previously described (Romano-Bertrand et al., 2012). PCR amplifications were checked by DNA electrophoresis in 1.5% agarose gels containing ethidium bromide and visualized under ultraviolet light. Sterile DNA-free water used for mosquito preparations was used in negative control for each series of DNA extraction and PCR. The successfully amplified products were sequenced with primer 27f, on an ABI 3730xl sequencer (Cogenics, Meylan, France). Each sequencing chromatograph was visually inspected and corrected.

### Independent-Culture Analysis by 16S PCR-Temporal Temperature Gel Electrophoresis (PCR-TTGE)

Whole DNA was extracted from 100 μl of mosquito abdomen suspension using the Master Pure Gram Positive DNA purification kit as recommended by the supplier (Epicentre Biotechnologies, Madison, WI, USA). The purified and raw DNAs were kept at -80°C before further analyses. Because of low bacterial load in mosquito’s samples, a nested-PCR approach was necessary to obtain a sufficient PCR amplification (Romano-Bertrand et al., 2012). The first PCR amplified almost entirely the 16S rRNA gene using the 27f and 1492r primers as previously described (Romano-Bertrand et al., 2012). Then, products of the first PCR were used as template for the amplification of the V2–V3 hyper variable region using the FastStart high fidelity PCR system (Roche apply science) and the primers HDA1 with GC-clamp and HDA2 (Ogier et al., 2002; Roudiere et al., 2009). Again, sterile DNA-free water was used as negative control for each series of nested-PCR. If positive signal was observed, controls were analyzed in parallel of samples by TTGE migration. TTGE profiles of samples and controls were then compared. Positive controls, consisting on decreasing inoculums of *Elizabethkingia anophelis* (which is especially retrieved from mosquitos samples), were used to validate the analysis, from the DNA extraction and throughout the whole Temporal Temperature Gel Electrophoresis (TTGE) process. TTGE migration and TTGE bands sequencing were performed as described in Ngo et al. (2015).

### Sequence Analysis, Species Identification, and OTU Affiliation

The sequences were analyzed by comparison with Genbank^1^ and Ribosomal Databases Project 2 (RDPII)^2^ using Basic Local Alignment Search Tool (BLAST) and Seqmatch programs, respectively. The sequence with the highest percentage was used for OTU affiliation as previously described by Ngo et al. (2015). Briefly, a sequence was affiliated to a species-level OTU when the percent of sequence similarity with the species type strain was above 99.0% (Drancourt et al., 2000). Below 99.0%, the sequence was affiliated to the genus of the reference sequence with the highest percentage. When several species reference sequences match equally, affiliation was done to the genus level or to a group of species if relevant. For example, sequence with 99.5% in similarity to both *Aeromonas caviae* and *A. hydrophila* was only assigned to the genus *Aeromonas*. The same rule was applied for the taxonomic level higher than the genus level if necessary. The 16S rRNA gene sequences from all cultured bacteria are available in GenBank (accession numbers KX449232–KX449311). Sequences from TTGE band are available by contacting corresponding author or by consulting the Supplementary Fasta File.

### Diversity Index Calculation and Statistical Analysis

The microbiota diversity for each species of *Anopheles* was estimated by the calculation of crude diversity index (DI, number of different OTUs; Romano-Bertrand et al., 2012, 2014), Shannon–Wiener DI (H) and Simpson’s index (D; Gafan et al., 2005; Ledder et al., 2007; Romano-Bertrand et al., 2014).

The 100 *Anopheles* specimens were classified into the Dirus Complex or into the Funestus and Maculatus Groups according to their species identification. The bacterial diversity scores (DI, H, and D) for the different *Anopheles* species, complex and groups were then calculated.

## Results

### *Anopheles* Identification

**FIGURE 1:**
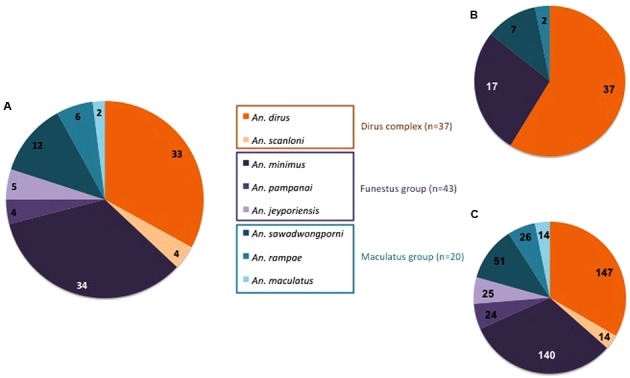
**Distribution according to *Anopheles* species, complex and group of (A)** mosquitoes specimens, **(B)** bacterial strains, and **(C)** TTGE bands.

Specific PCR assays identified eight *Anopheles* species among the 100 specimens collected in Binh Phuoc Province. Out of 100, 37 specimens belonged to the Dirus Complex including *An. dirus* (*n* = 33) and *An. scanloni* (*n* = 4), 43 to the Funestus Group with *An. minimus* (*n* = 34), *An. jeyporiensis* (*n* = 5), and *An. pampanai* (*n* = 4), and 20 to the Maculatus Group with *An. sawadwongporni* (*n* = 12), *An. rampae* (*n* = 6), and *An. maculatus* (*n* = 2; **Figure 1A**).

### Bacterial Diversity in the Abdomen of *Anopheles* Adults

The abdomens of 100 *Anopheles* were analyzed using both bacterial culture and PCR-TTGE methods. Only a few negative controls presented weak amplification bands in agarose gel after nested-PCR but these bands were never observed after TTGE migration, permitting to validate the profiles for mosquitoes’ samples. The regular positive controls highlighted a sensitivity of detection comprised between 10^1^ and 10^2^ bacterial inoculums throughout the TTGE process (data not shown). Bacteria were cultured or detected in 98% of the *Anopheles* specimens, the two negative specimens belonged to species *An. minimus* and *An. scanloni*. A total of 63 bacterial strains from 22 specimens positive by culture and 441 TTGE bands from 98 specimens positive by PCR were obtained. Distributions of strains and TTGE bands according to *Anopheles* species are shown in **Figures 1B,C**, respectively.

**FIGURE 2:**
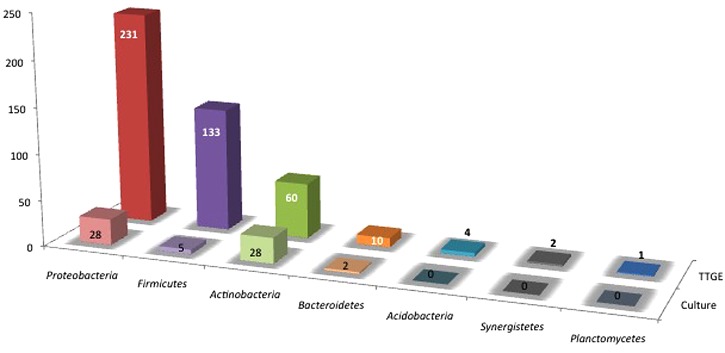
**Distribution of TTGE bands and bacterial isolates according to phylum**.

**FIGURE 3:**
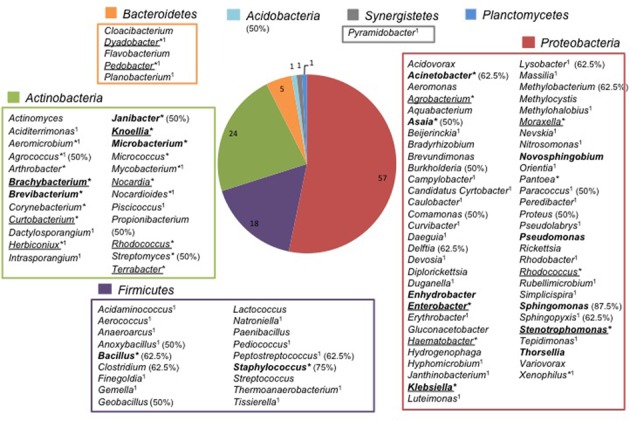
**Repertory of genus-level OTUs according to phylum in adult *Anopheles* gut microbiota**. ^∗^Cultivable genera; Genera only retrieved in culture are underlined; ^1^Genera identified in *Anopheles* abdomen for the first time; Genera previously found in *Anopheles* from Vietnam in bold face (Ngo et al., 2015); In parenthesis, the percent (50% and higher) of *Anopheles* species carrying genera frequently found in Binh Phuoc *Anopheles* microbiota.

Both strains and 393 TTGE bands were further analyzed by 16S rRNA gene PCR and sequenced. The remaining 48 TTGE bands were identified by comparison with another co-migrating band on the same TTGE gel. Results were presented in Supplementary Tables. Strains and PCR-TTGE bands were affiliated to seven phyla, the most represented being *Proteobacteria*, *Firmicutes*, and *Actinobacteria* (**Figure 2**). Considering OTUs, 193 bacterial OTUs corresponding to 190 genera-level OTUs (including 85 OTUs for which identification was performed until species-level), one family-level and two phyla-level OTUs. The sequences obtained from 3 TTGE bands were not accurate enough to be affiliated to the genus level, probably due to mixed DNA amplified and sequenced from co-migrating bands; one of them was affiliated to the Phylum *Acidobacteria* and two others to the Family *Planctomycetaceae* (Phylum *Planctomycetes*). The OTU richness by phylum is summarized in **Figure 3**. *Proteobacteria* was the most diversified phylum with a richness corresponding to 57 genera-level OTUs, followed by *Actinobacteria* and *Firmicutes* with, respectively, 24 and 18 genera-level OTUs.

Only 32 from the 190 genera-level OTUs were retrieved in culture, mainly from *Actinobacteria* with 18 cultivable genera (56%). Out of these 32 cultivable OTUs, 15 (7.9%) were not retrieved by the molecular approach (genera underlined in **Figure 3** and Supplementary Tables).

In this study, molecular approach allowed the detection of more bacteria than culture, although culture improved the description of bacterial richness in the *Anopheles* abdomen microbiota and should be considered as complementary to PCR-based methods. The *Actinobacteria* richness was particularly widen thanks to culture.

### Bacterial Richness and Diversity of the Abdomen Microbiota According to the *Anopheles* Species

**FIGURE 4:**
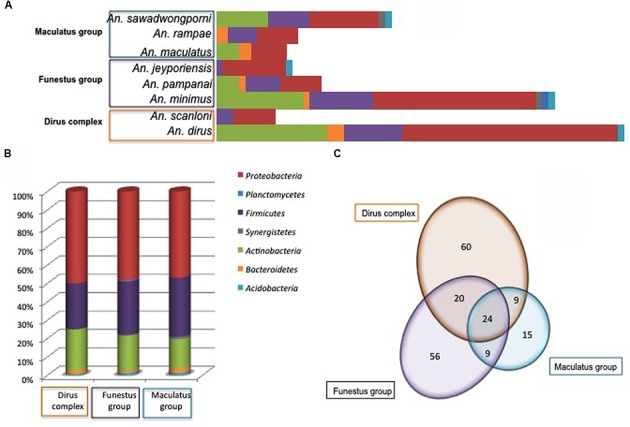
**Number of different OTUs per phylum and *Anopheles* species (A)**, percent of OTUs in every phyla **(B)**, and distribution of shared and specific OTUs (Venn diagram in **C**) according to *Anopheies* complex and groups. The phyla color code is given for **(A)** and **(B)**.

Bacterial diversity according to *Anopheles* species was displayed in **Figure 4A**. No obvious link was observed between *Anopheles* complex or groups and microbiota diversity. However, the number of specimens in several *Anopheles* species is too low for robust conclusions, only tendencies can be drawn. Amongst the three major phyla, *Firmicutes* and *Proteobacteria* were present in all *Anopheles* species, while *Actinobacteria* were absent from *An. jeyporiensis*, *An. scanloni*, and *An. rampae* (**Figure 4**). *An. minimus*, considered as one of the primary vector of *Plasmodium* in Southeast Asia, including Vietnam, was the sole species colonized by members of the seven bacterial phyla. Two other malaria vectors well represented in the collection, *An. dirus* and *An. sawadwongporni*, were each colonized by five bacterial phyla (**Figure 4A**). Despite the low number of specimens (*n* = 6) in this species, *An. rampae* presented a particular diversity profile with the replacement of *Actinobacteria* by *Bacteroidetes* regarding other *Anopheles* species. Nevertheless, the phylum richness appeared more dependent to the number of specimens in a species of *Anopheles* than to the *Anopheles* complex and groups (**Figures 4A,B**). Indeed, if *Anopheles* complex or group were considered, the phyla distribution in percent was very similar among them (**Figure 4B**).

However, at the OTU level the distribution varied according *Anopheles* complex and groups (**Figure 4C**). Among the 193 OTUs, 113, 109, and 57 OTUs were identified within the Dirus Complex, the Funestus and Maculatus Groups, respectively (**Figure 4C**). Out of them, 24 OTUs were shared by the three *Anopheles* taxa, 20 OTUs were shared by the Dirus Complex and the Funestus Group, and nine OTUs were commonly identified either between the Funestus and Maculatus Groups, or the Dirus Complex and Funestus Group (**Figure 4C**).

*Anopheles* abdomen microbiota diversity quantified by the mean index and scores was presented in **Table 1**. The average DI per specimen reached 4.843. *An. maculatus* (DI = 7.0) and *An. jeyporiensis* (DI = 6.0) microbiota were the most diverse, whereas *An. minimus* displayed lowest diversity (DI = 4.429) (**Table 1**). Considering both bacterial richness and equitability of the OTUs within *Anopheles* species, the average Shannon–Wiener score (H) for all species was 5.569. *An. minimus* had the higher *H* score (*H* = 6.213) indicating that the numbers of OTUs are evenly distributed between all specimens (**Table 1**). The Simpson index (D) takes into account both bacterial species richness and an evenness of abundance among the bacterial species present, and is inversely proportional to the diversity (DI). For all *Anopheles* species, except *An. sawadwongporni* (*D* = 0.308), the *D*-values were low ranging between 0 and 0.082, suggesting high diversities (**Table 1**). However, these results should be taken with caution because of the low number of specimens for *An. scanloni* (*n* = 3), *An. jeyporiensis* (*n* = 5), *An. pampanai* (*n* = 4), *An. maculatus* (*n* = 2), and *An. rampae* (*n* = 6).

**Table 1:** Number of OTUs and diversity indexes for each species of *Anopheles*.

Complex or group	Species	Number of different OTUs	Dl (mean ±*SD*)	H (mean ±*SD*)	D (mean ±*SD*)
Dirus complex	*An. dirus*	110	5.176 ( ± 2.276)	4.446 ( ± 0.024)	0.011 ( ± 0.001)
	*An. scanloni*	13	4.667 ( ± 2.517)	2.656 ( ± 0.025)	0.013 ( ± 0.004)
	All	115	5.135 ( ± 2.263)	4.585 ( ± 0.022)	0.009 ( ± 0.000)
Funestus group	*An. minimus*	94	4.429 ( ± 1.852)	6.213 ( ± 0.039)	0.042 ( ± 0.005)
	*An. pampanai*	19	6.000 ( ± 2.708)	3.327 ( ± 0.042)	0.035 ( ± 0.004)
	*An. jeyporiensis*	19	5.000 ( ± 2.449)	3.327 ( ± 0.042)	0.035 ( ± 0.004)
	All	110	4.636 ( ± 2.001)	6.488 ( ± 0.028)	0.022 ( ± 0.001)
Maculatus group	*An. maculatus*	14	7.000 ( ± 1.414)	2.639 ( ± 0.000)	0 ( ± 0)
	*An. sawadwongporni*	41	4.462 ( ± 2.145)	4.552 ( ± 0.042)	0.308 ( ± 0.018)
	*An. rampae*	19	4.467 ( ± 1.506)	3.554 ( ± 0.058)	0.082 ( ± 0.009)
	All	60	4.762 ( ± 1.998)	4.798 ( ± 0.547)	0.012 ( ± 0.000)
	All	193	4.843 ( ± 2.098)	5.569 ( ± 0.011)	0.003 ( ± 0.000)

### The Core Microbiota of Vietnam *Anopheles* Is Limited

Three *Firmicutes* genera, *Staphylococcus, Bacillus*, and *Clostridium*, were the most prevalent in the abdomen microbiota of Binh Phuoc *Anopheles*, respectively, identified in 29.6, 23.5, and 21.4% of the specimens. Besides, *Propionibacterium*, *Peptostreptococcus, Acinetobacter, Burkholderia, Comamonas, Delftia, Lysobacter*, and *Sphingomonas* were detected in more than 10% of the specimens, with frequencies ranging from 10.2 to 18.4%. The Family *Enterobacteriaceae* (*Enterobacter, Klebsiella, Pantoea*, and *Proteus*) was detected in 15.3% of the specimens. The other 90 genera were found at frequencies lower than 10%, including 81 genera detected in only 1–4 samples (<5%), certainly due to the important bacterial diversity in the mosquito microbiota (Supplementary Table 1).

The most represented genera in Binh Phuoc *Anopheles* microbiota were *Staphylococcus* and *Sphingomonas* (Supplementary Table 1 and **Figure 3**). Six other genera retrieved at lower frequencies were newly identified in our study: *Agrococcus*, *Anoxybacillus*, *Peptostreptococcus*, *Lysobacter*, *Paracoccus*, and *Sphingopyxis* (Supplementary Table 1 and **Figure 3**). Finally, a total of 21 genera were detected in more than 4 of 8 *Anopheles* species but these genera were present in <30% of the mosquito specimens confirming that the high bacterial diversity in Binh Phuoc *Anopheles* impaired the definition a core microbiota. Nevertheless, the presence of some frequent genera in Binh Phuoc *Anopheles* microbiota that were not yet described in *Anopheles* pan-microbiota, suggested the existence of specific microbiota according to the geographic region probably due to a strong influence of local environmental conditions.

Concerning the pan-microbiota of the whole genus *Anopheles*, meta-analysis of recent studies (Gendrin and Christophides, 2013; Manguin et al., 2013; Minard et al., 2013; Sharma et al., 2014; Villegas and Pimenta, 2014; Ngo et al., 2015) and bibliography databases survey for older publications, indicated that 51 bacterial genera (48.6%) were probably detected herein for the first time in *Anopheles* (1 in **Figure 3** and Supplementary Table 1). These newly described genera could be slightly overestimated due to the meta-analysis approach and the comparison with non-fully exhaustive data but it clearly suggested that the overall richness of *Anopheles* microbiome is far from being fully explored. This is particularly true in Southeast Asia where very little work has been done on this topic (Gendrin and Christophides, 2013; Ngo et al., 2015).

In comparison with a previous study on the microbiota diversity in specimens from Dak Nong Province, Vietnam (Ngo et al., 2015), 17 genera (in bold in **Figure 3**) were common in *Anopheles* species from both provinces. Most of these genera were found at frequencies below 10% in the *Anopheles* specimens from Binh Phuoc Province, except four genera: *Sphingomonas* (13.3%), *Acinetobacter* (18.4%), *Bacillus* (23.5%), and *Staphylococcus* (29.6%). The genus *Acinetobacter* was detected in more than half *Anopheles* species in both Binh Phuoc and Dak Nong *Anopheles* microbiota. It could be considered as the sole genus forming the core microbiota of Vietnam *Anopheles*.

### Characterization of the Abdomen Microbiota of *Plasmodium* Naturally Infected *Anopheles* Specimens

Two specimens were naturally infected by *P. vivax*, out of the 486 *Anopheles* specimens collected in Binh Phuoc Province (Ngo et al., 2014). They belonged to the species *An. dirus* and *An. pampanai*. The abdomen bacterial microbiota of these two specimens was described by PCR-TTGE only, because cultured isolates were not obtained from these two infected mosquitoes. The sequence analysis of seven TTGE bands showed the presence of six bacterial taxa: *Acinetobacter* sp., *Geobacillus* sp., *Luteimonas* sp., *Methylobacterium* sp., *Rubellimicrobium* sp., and *Sphingomonas* sp., among which five (83.3%) belonged to *Proteobacteria* and only one, *Geobacillus* sp., to *Firmicutes*. The genus *Acinetobacter*, *Geobacillus*, *Methylobacter*, and *Sphingomonas* belonged to the core^50%^ microbiota and were also retrieved from other non-infected specimens of *Anopheles*, whereas *Luteimonas* was only retrieved from the infected *An. pampanai* specimen, and *Rubellimicrobium* from both the infected *An. dirus* and one specimen of *An. rampae* (Supplementary Table 1).

## Discussion

The description of microbiota in vector arthropods presented increasing interest because of potential role in vector competence. Protective role of *Anopheles* bacterial microbiota against *Plasmodium* infections has been demonstrated because clearing midgut microbiota by antibiotic treatment resulted in enhanced *Plasmodium* infections rate (Beier et al., 1994; Favia et al., 2007; Dong et al., 2009; Rodrigues et al., 2010). Similarly but in semi-natural settings, antibiotics in ingested blood enhanced the susceptibility of *An. gambiae* to *Plasmodium* infection and increasing mosquito fitness through higher survival and fecundity. Moreover, the presence of antibiotics in the blood of malaria-infected people was identified as a risk for increased disease transmission (Gendrin et al., 2015). In addition, the experimental co-infections of bacteria with *Plasmodium* have showed the reduction of the number of developing oocysts in the mosquito midgut, both in laboratory and field conditions (Pumpuni et al., 1993; Lowenberger et al., 1999; Gonzalez-Ceron et al., 2003; Dong et al., 2009; Meister et al., 2009; Cirimotich et al., 2011). More recently, it was observed that expansion of midgut microbiota by negative regulation of *An. gambiae* immune response increased susceptibility to *Plasmodium* as observed before for microbiota depletion (Dennison et al., 2015). Finally, it’s probably the microbiota equilibrium that confers resistance to *Plasmodium* infection. Then, the development of new approaches in malaria control needs the description of the microbiota of wild *Anopheles* species and its variations (Rani et al., 2009; Boissière et al., 2012; Chavshin et al., 2012; Osei-Poku et al., 2012; Gendrin and Christophides, 2013; Manguin et al., 2013; Ngo et al., 2015).

As previously done (Ngo et al., 2015), we present herein a combined approach associating molecular detection and culture that improve the performance of each separate method. About one third of the bacterial genera detected in this study were obtained by culture, thus providing a valuable collection of isolates for further experiments such as experimental challenges (Ramirez et al., 2014) and in paratransgenesis projects (Chavshin et al., 2014). Due to the low resolution of TTGE fingerprints when the number of band is high, PCR-TTGE is only suitable for the description of microbiota with relative low richness as demonstrated for human neonatal microbiota (Jacquot et al., 2011) and surgical microbiota (Romano-Bertrand et al., 2014), but also for the description of mosquito microbiota (Manguin et al., 2013; Ngo et al., 2015). By this mean, we previously described most of the known repertory of bacterial taxa found in Asian *Anopheles* as shown by Villegas and Pimenta (2014). TTGE fingerprinting gave a good description of majority bacterial populations in mosquito microbiota, with a rather good sensitivity, as demonstrated by the extent of the taxonomic richness described herein compared to other studies even those which used Next Generation Sequencing (NGS; Villegas and Pimenta, 2014). Moreover, the use of PCR-TTGE in this study allowed a comparison with previous studies in Vietnam (Ngo et al., 2015), but also in Thailand (Manguin et al., 2013). NGS is now becoming a reference method for microbiota description and knowledge of mosquito-associated bacteria would likely benefit from studies by NGS. Because NGS provide huge amounts of data sometimes partially exploited, preliminary and comparative studies by TTGE or other community fingerprinting approaches allow asking scientific questions to be further in-depth explored by NGS.

The *Anopheles* collection of 100 wild specimens collected in Binh Phuoc Province was colonized by a great diversity of bacteria with, to our knowledge, 51 genera not yet described in anophelines. Thereby, this study widens the description of the *Anopheles* pan-microbiota. The huge diversity of *Anopheles* associated bacteria contrasts the low richness observed per specimen. This result is similar to that shown by Osei-Poku et al. (2012) in different mosquito genera, including *Anopheles*, in which bacterial richness per specimen is very low, nearly always dominated by a small number of taxa. This low richness could explain that only 78% of the specimens are positive in culture. Other reasons could be the trapping of some bacteria in the *Anopheles* tissues despite the preparative crushing step, the presence of viable but non-cultivable bacteria and the limited panel of culture medium used in the study. Culturomics approaches (Tandina et al., 2016) with the use of large panel of culture media and conditions could enhance the culture yield in next studies.

Within the bacteria detected in *Anopheles* specimens from Binh Phuoc, approximately half of the genera have previously been identified in the intra-abdominal microbiota of *Anopheles* populations from Thailand (Manguin et al., 2013), Vietnam (Manguin et al., 2013; Ngo et al., 2015), Cameroon (Boissière et al., 2012), Iran (Chavshin et al., 2012), India (Rani et al., 2009), or in reviews (Gendrin and Christophides, 2013; Minard et al., 2013; Hughes et al., 2014; Villegas and Pimenta, 2014).

Our results also showed that there is no link between bacterial diversity and host species or group of host species collected in Binh Phuoc Province. This result is in accordance with previous reports that rather showed the influence of the local environment of the sampling site, environment that gives a specific bacterial profile to mosquito specimens (Boissière et al., 2012; Manguin et al., 2013; Buck et al., 2016). The microbiota diversity of host species will also be significantly influenced by the origin of specimens like wild *versus* laboratory reared *Anopheles* mosquitoes (Chavshin et al., 2012). For instance, the selective pressure of laboratory conditions may limit bacteria acquisition at both larval and adult stages resulting in great reduction or nil cultivable bacteria in mosquito midgut (Riehle and Jacobs-Lorena, 2005; Favia et al., 2007; Gusmão et al., 2010). Studies on microbiota of *An. gambiae* larvae and pupae showed that only subset of bacteria from the aquatic habitat were able to inhabit the mosquito gut (Wang et al., 2011). The same influence of environment on *Anopheles* microbiota is likely to occur during the terrestrial life of adults. Considering the repertory of bacterial genera presented in **Figure 3**, the presence of soil- and plant-associated bacteria in *Anopheles* microbiota is probable. For instance, *Agrococcus*, *Janibacter*, *Streptomyces, Bacillus* (and related genera), and *Lysobacter* are classical soil inhabitants, whereas *Asaia*, *Burkholderia*, *Comamonas*, *Delftia*, and *Sphingomonas* and *Methylobacterium* are frequently associated with plants. In addition, several prevalent bacterial genera are associated with animal or human microbiota, mainly to cutaneous microbiota: *Propionibacterium*, *Staphylococcus*, *Peptostreptococcus*, *Clostridium*, and *Acinetobacter*. These results suggest that bacteria from diverse origins could colonize adult *Anopheles*.

Interestingly, the *Anopheles* species from Binh Phuoc Province contained members of the genus *Enterobacter* sp. recently isolated from wild *An. gambiae* from Zambia. *Enterobacter* sp. received lot of attention due its anti-*Plasmodium* effect through the production of reactive oxygen species that directly target *Plasmodium* parasites in the midgut of *An. gambiae* (Cirimotich et al., 2011). More recently, another bacteria, *Chromobacterium Csp_P*, has been described as responsible of vectors refractoriness to *P. falciparum* and dengue virus (Ramirez et al., 2014). Then, further studies on mosquito microbiota should provide new insights on pathogens transmission and might open new ways to prevent vector-borne diseases.

Recently published studies have described new bacterial species, first isolated from *Anopheles* mosquitoes, such as *E. anophelis* (Kampfer et al., 2011), *Thorsellia anophelis* (Kampfer et al., 2006a), and *Janibacter anophelis* (Kampfer et al., 2006b). The latter species was accurately identified herein after bacterial culture and the species *J. terrae* described in soil was also found in Binh Phuoc *Anopheles*. Bacterial identification to the species level or below, defining variants within a bacterial species (genovar, genomovar, biovar, etc.), is needed to determine whether strictly identical bacterial populations are shared between environment, host and vector, or if *Anopheles*-associated bacteria are specific.

As a tendency to be confirmed, we also described herein the microbiota of two *Anopheles* (*An. dirus* and *An. pampanai*) naturally infected by *P. vivax*. The genus *Acinetobacter* solely was common to the two infected specimens but it was also one of the most prevalent bacterial species forming *Anopheles* microbiota. Indeed, it was frequently identified in the microbiota of both Asian and African anopheline species (Cameroon, Iran, India, Kenya, Mali, and Vietnam; Straif et al., 1998; Rani et al., 2009; Chavshin et al., 2012). Nevertheless, there is no available report on the implication of this genus for the development of *Plasmodium* in *Anopheles* and the interaction between this bacterial genus and malarial parasites in *Anopheles* might be studied.

The huge diversity of the microbial world even within a single species urges to overpass genus or species level for microbiota descriptions. Indeed, it is likely that the informative taxonomic level for host and microbes interactions is the subspecies level. Such a precise characterization needs availability of bacterial strains, highlighting again the interest of culture in microbiota studies. Moreover, the phenotype of bacterial strains could be of great interest to understand mechanisms involved in bacteria/*Plasmodium*/*Anopheles* interactions. This study provides 51 strains available for further experiments. Moreover, to the niche level, functional metagenomic and metabolomic approaches should be undertaken to determine microbiota conditions that favor or not vector competence.

## Author Contributions

CN made the bacterial analyses (bacterial cultures and molecular analyses), and results’ interpretation. SR-B helped for DNA sequencing, bacterial identification, and results’ interpretation. SM contributed to the study design, the specimens collection and the results’ interpretation. EJ-B supervised the study design, the bacterial analyses, and the results’ interpretation. All the authors contributed to the manuscript redaction.

## Conflict of Interest Statement

The authors declare that the research was conducted in the absence of any commercial or financial relationships that could be construed as a potential conflict of interest.

## Footnotes

1 http://www.ncbi.nlm.nih.gov/

2 http://rdp.cme.msu.edu/
