# Supplementary material for: Diversity of the Bacterial Microbiota of Anopheles Mosquitoes from Binh Phuoc Province, Vietnam
Source: Front Microbiol. 2016 Dec 23;7:2095. doi: 10.3389/fmicb.2016.02095 (PMC5181100; doi:10.3389/fmicb.2016.02095)
Supplement: Supplementary file 1 [file Table_1.docx]

**Supplementary Material**

**Diversity of the bacterial microbiota of Anopheles mosquitoes from Binh Phuoc Province, Vietnam**

**Chung Thuy Ngo, Sara Romano-Bertrand*, Sylvie Manguin and Estelle Jumas-Bilak**

*** Correspondence:** Sara Romano-Bertrand: sara.romano-bertrand@univ-montp1.fr

**Supplementary Table 1.** 105 bacterial genera detected in abdomen of Anopheles species from Binh Phuoc Province, Vietnam, with the number of specimens carrying each genus.

This table compiles all the genus of bacteria that have been detected for all specimens according to the Anopheles species and corresponding frequencies of identification. Total number of bacterial genera per Anopheles species is given at the bottom of the table.

* Cultivable genera, genera only retrieved in culture are underlined. (1) Genera newly identified in Anopheles mosquitoes according to the recent reviews (Gendrin and Christophides, 2013; Manguin et al., 2013; Minard et al., 2013; Segata et al., 2016; Sharma et al., 2014; Villegas and Pimenta, 2014).

| **Phyla** | **Bacterial family/genera** | **Dirus Complex** | | **Funestus Group**  **(n=42)** | | | **Maculatus Group (n=20)** | | | **Frequencies of identification** |
| --- | --- | --- | --- | --- | --- | --- | --- | --- | --- | --- |
|  |  | **(n=36)** | |  |  |  |  |  |  |  |
|  |  | **An. dirus** | **An. scanloni** | **An. minimus** | **An. jeyporiensis** | **An. pampanai** | **An. maculatus** | **An. rampae** | **An. sawadwongporni** |  |
|  |  | **(n = 33)** | **(n = 3)** | **(n = 33)** | **(n = 5)** | **(n = 4)** | **(n = 2)** | **(n = 6)** | **(n = 12)** |  |
| Acidobacteria | unknown genera | 1 |  | 1 | 1 |  |  |  | 1 | 4.1% |
| Actinobacteria | Actinomyces (1) | 1 |  | 1 |  |  |  |  |  | 2% |
|  | Aciditerrimonas (1) | 1 |  | 1 |  | 1 |  |  |  | 3% |
|  | Aeromicrobium * (1) | 1 |  |  |  |  |  |  |  | 1% |
|  | Agrococcus * (1) | 3 |  | 2 |  |  | 1 |  | 1 | 7.1% |
|  | Arthrobacter * (1) | 1 |  |  |  |  |  |  | 1 | 2% |
|  | Brachybacterium * | 1 |  |  |  |  |  |  |  | 1% |
|  | Brevibacterium * | 1 |  |  |  |  |  |  |  | 1% |
|  | Corynebacterium * | 3 |  | 3 |  |  | 1 |  |  | 7.1% |
|  | Curtobacterium * | 1 |  |  |  |  |  |  |  | 1% |
|  | Dactylosporangium (1) |  |  | 1 |  |  |  |  |  | 1% |
|  | Herbiconiux * (1) |  |  | 1 |  |  |  |  |  | 1% |
|  | Intrasporangium (1) | 1 |  |  |  |  |  |  |  | 1% |
|  | Janibacter * | 3 |  | 1 |  | 1 |  |  | 1 | 6.1% |
|  | Knoellia * | 1 |  | 1 |  |  |  |  | 1 | 3% |
|  | Microbacterium * | 3 |  | 1 |  |  |  |  | 1 | 5.1% |
|  | Micrococcus * | 1 |  | 2 |  |  |  |  | 1 | 4.1% |
|  | Mycobacterium * (1) | 1 |  | 1 |  |  |  |  | 1 | 3% |
|  | Nocardia * (1) |  |  | 1 |  |  |  |  |  | 1% |
|  | Nocardioides * (1) |  |  | 1 |  |  |  |  | 1 | 2% |
|  | Piscicoccus (1) | 1 |  |  |  |  |  |  |  | 1% |
|  | Propionibacterium | 6 |  | 9 |  | 1 | 1 |  |  | 17.3% |
|  | Rhodococcus * | 1 |  |  |  |  |  |  |  | 1% |
|  | Streptomyces * | 4 |  | 3 |  | 1 | 1 |  |  | 9.2% |
|  | Terrabacter * |  |  |  |  |  |  |  | 1 | 1% |
| Bacteroidetes | Cloacibacterium | 1 |  |  |  | 1 |  | 1 |  | 3% |
|  | Dyadobacter * (1) | 1 |  |  |  |  |  |  |  | 1% |
|  | Flavobacterium |  |  | 1 |  |  |  |  |  | 1% |
|  | Pedobacter * (1) | 1 |  |  |  |  |  |  |  | 1% |
|  | Planobacterium (1) |  |  |  |  |  |  | 1 |  | 1% |
| Firmicutes | Acidaminococcus (1) |  |  | 1 |  |  |  |  |  | 1% |
|  | Aerococcus (1) |  |  | 2 |  |  |  |  |  | 2% |
|  | Anaeroarcus (1) | 2 |  |  |  |  |  |  |  | 2% |
|  | Anoxybacillus (1) | 4 |  | 1 |  |  |  | 1 | 1 | 7.1% |
|  | Bacillus * | 11 | 1 | 9 | 1 | 1 |  |  |  | 23.5% |
|  | Clostridium | 3 |  | 9 |  | 2 |  | 3 | 4 | 21.4% |
|  | Finegoldia (1) |  |  |  |  | 1 |  |  | 1 | 2% |
|  | Gemella (1) |  |  | 1 |  |  | 1 |  |  | 2% |
|  | Geobacillus | 3 |  | 1 |  | 1 |  |  | 1 | 6.1% |
|  | Lactococcus |  |  |  |  |  |  |  | 1 | 1% |
|  | Natroniella (1) | 1 |  | 1 |  |  |  |  |  | 2% |
|  | Paenibacillus |  |  |  |  |  |  | 1 |  | 1% |
|  | Pediococcus (1) | 1 |  |  |  |  |  |  |  | 1% |
|  | Peptostreptococcus (1) | 2 |  | 2 |  | 1 |  | 2 | 3 | 10.2% |
|  | Staphylococcus* | 7 | 2 | 12 |  | 2 |  | 3 | 3 | 29.6% |
|  | Streptococcus | 1 | 1 |  |  |  |  |  |  | 2% |
|  | Thermoanaerobacterium (1) |  |  |  |  |  | 1 |  |  | 1% |
|  | Tissierella (1) |  |  | 2 |  |  |  |  |  | 2% |
| Planctomycetes | Planctomycetaceae |  |  | 1 |  |  |  |  |  | 1% |
| Proteobacteria | Acidovorax | 3 |  | 1 |  |  |  |  |  | 4.1% |
|  | Acinetobacter * | 7 |  | 5 |  | 3 | 1 |  | 2 | 18.4% |
|  | Aeromonas |  |  | 2 |  |  |  |  |  | 2% |
|  | Agrobacterium * | 3 |  |  |  |  |  |  |  | 3% |
|  | Aquabacterium |  |  |  |  |  |  | 1 | 1 | 2% |
|  | Asaia * | 1 |  | 5 |  |  |  | 1 | 1 | 8.2% |
|  | Beijerinckia (1) | 1 |  |  |  | 1 |  |  |  | 2% |
|  | Bradyrhizobium | 1 |  |  |  |  |  |  |  | 1% |
|  | Brevundimonas | 1 |  | 1 | 1 |  |  |  |  | 3% |
|  | Burkholderia | 3 | 1 | 3 | 3 |  |  |  |  | 10.2% |
|  | Campylobacter (1) | 1 |  |  |  |  |  |  |  | 1% |
|  | Candidatus Cyrtobacter (1) |  |  | 1 |  |  |  |  |  | 1% |
|  | Caulobacter (1) |  |  |  | 2 |  |  |  |  | 2% |
|  | Comamonas | 8 | 1 | 4 |  |  |  |  | 2 | 15.3% |
|  | Curvibacter (1) |  | 1 |  |  |  |  |  |  | 1% |
|  | Daeguia (1) | 1 |  |  |  |  |  |  |  | 1% |
|  | Delftia | 7 | 1 | 5 | 2 |  | 1 |  |  | 16.3% |
|  | Devosia (1) | 1 |  | 1 |  |  |  |  |  | 2% |
|  | Diplorickettsia | 1 |  |  |  |  |  |  |  | 1% |
|  | Duganella (1) |  |  |  |  |  | 1 |  |  | 1% |
|  | Enhydrobacter |  |  | 1 |  |  |  |  |  | 1% |
|  | Enterobacter * | 1 |  |  |  |  |  |  |  | 1% |
|  | Erythrobacter (1) |  |  |  | 1 |  |  |  |  | 1% |
|  | Gluconacetobacter | 3 |  |  |  |  |  |  |  | 3% |
|  | Haematobacter * | 1 |  |  |  |  |  |  |  | 1% |
|  | Hydrogenophaga |  |  |  | 1 |  |  |  |  | 1% |
|  | Hyphomicrobium (1) |  |  | 1 |  |  |  |  |  | 1% |
|  | Janthinobacterium (1) | 1 |  | 1 |  |  |  |  |  | 2% |
|  | Klebsiella * | 1 |  |  |  |  |  |  |  | 1% |
|  | Luteimonas (1) |  |  |  |  | 1 |  |  |  | 1% |
|  | Lysobacter (1) | 1 |  | 4 |  | 1 |  | 4 | 4 | 14.3% |
|  | Massilia (1) | 1 |  | 2 |  |  |  |  | 1 | 4.1% |
|  | Methylobacterium | 1 | 1 | 1 |  | 1 |  |  | 2 | 6.1% |
|  | Methylocystis |  |  | 1 |  |  |  |  |  | 1% |
|  | Methylohalobius (1) | 1 |  |  |  |  |  |  | 1 | 2% |
|  | Moraxella * |  |  | 1 |  |  |  |  |  | 1% |
|  | Nevskia (1) | 1 |  | 1 |  |  |  |  |  | 2% |
|  | Nitrosomonas (1) | 1 |  |  |  |  |  |  |  | 1% |
|  | Novosphingobium | 1 |  |  | 2 |  |  |  |  | 3% |
|  | Orientia (1) |  |  | 1 |  |  |  |  |  | 1% |
|  | Pantoea * | 3 |  | 2 |  |  |  |  |  | 5.1% |
|  | Paracoccus (1) | 2 |  | 2 |  |  | 1 | 2 |  | 7.1% |
|  | Peredibacter (1) |  |  | 1 |  |  |  |  |  | 1% |
|  | Proteus | 3 |  | 3 |  | 1 | 1 |  |  | 8.2% |
|  | Pseudolabrys (1) | 1 |  |  |  |  |  |  |  | 1% |
|  | Pseudomonas |  |  |  | 2 |  |  |  |  | 2% |
|  | Rickettsia |  |  | 1 |  |  |  |  |  | 1% |
|  | Rhodobacter (1) | 1 |  |  |  |  |  |  |  | 1% |
|  | Rhodococcus * | 1 |  |  |  |  |  |  |  | 1% |
|  | Rubellimicrobium (1) | 1 |  |  |  |  |  | 1 |  | 2% |
|  | Simplicispira (1) |  |  |  | 1 |  |  |  |  | 1% |
|  | Sphingomonas | 2 | 1 | 3 | 1 | 2 |  | 2 | 2 | 13.3% |
|  | Sphingopyxis (1) | 1 |  | 1 |  |  | 1 | 1 | 2 | 6.1% |
|  | Stenotrophomonas * | 1 |  |  |  |  |  |  | 1 | 2% |
|  | Tepidimonas (1) | 2 |  | 1 |  |  |  |  |  | 3% |
|  | Thorsellia |  | 1 |  |  |  |  |  |  | 1% |
|  | Variovorax (1) |  |  |  | 1 |  |  |  | 1 | 2% |
|  | Xenophilus * (1) | 1 |  |  |  |  |  |  |  | 1% |
| Synergistetes | Pyramidobacter (1) |  |  | 1 |  |  |  |  | 1 | 2% |
| **Number of genera** | | **69** | **10** | **56** | **12** | **18** | **12** | **14** | **29** |  |

**Supplementary Table 2.** 51 bacterial isolates identifications according to the Anopheles species.

| **Phyla** | **Bacterial isolates identification** | **Dirus Complex** | | **Funestus Group**  **(n=42)** | | | **Maculatus Group (n=20)** | | |
| --- | --- | --- | --- | --- | --- | --- | --- | --- | --- |
|  |  | **(n=36)** | |  |  |  |  |  |  |
|  |  | **An. dirus** | **An. scanloni** | **An. minimus** | **An. jeyporiensis** | **An. pampanai** | **An. maculatus** | **An. rampae** | **An. sawadwongporni** |
|  |  | **(n = 33)** | **(n = 3)** | **(n = 33)** | **(n = 5)** | **(n = 4)** | **(n = 2)** | **(n = 6)** | **(n = 12)** |
| Actinobacteria | Aeromicrobium kwangyangensis | 1 |  |  |  |  |  |  |  |
|  | Agrococcus terreus | 1 |  |  |  |  |  |  |  |
|  | Arthrobacter sp. | 1 |  |  |  |  |  |  |  |
|  | Corynebacterium callunae | 1 |  |  |  |  |  |  |  |
|  | Curtobacterium citreum | 1 |  |  |  |  |  |  |  |
|  | Herbiconiux solani |  |  | 1 |  |  |  |  |  |
|  | Janibacter anophelis |  |  | 1 |  |  |  |  |  |
|  | Janibacter terrae |  |  | 1 |  |  |  |  |  |
|  | Knoellia sinensis |  |  | 1 |  |  |  |  | 1 |
|  | Knoellia sp. | 1 |  |  |  |  |  |  |  |
|  | Microbacterium arborescens | 1 |  |  |  |  |  |  | 1 |
|  | Microbacterium lacus | 1 |  |  |  |  |  |  |  |
|  | Microbacterium phyllosphaerae | 1 |  |  |  |  |  |  |  |
|  | Micrococcus luteus | 1 |  |  |  |  |  |  |  |
|  | Micrococcus sp. |  |  |  |  |  |  |  | 1 |
|  | Micrococcus terreus |  |  | 1 |  |  |  |  |  |
|  | Mycobacterium fortuitum |  |  | 1 |  |  |  |  |  |
|  | Nocardia cyriacigeorgica |  |  | 1 |  |  |  |  |  |
|  | Nocardioides ganghwensis |  |  | 1 |  |  |  |  | 1 |
|  | Streptomyces capoamus | 1 |  |  |  |  |  |  |  |
|  | Streptomyces kunmingensis | 1 |  |  |  |  |  |  |  |
|  | Terrabacter tumescens |  |  |  |  |  |  |  | 1 |
| Bacteroidetes | Dyadobacter sp. | 1 |  |  |  |  |  |  |  |
|  | Pedobacter kribbensis | 1 |  |  |  |  |  |  |  |
| Firmicutes | Bacillus jeotgali |  |  | 1 |  |  |  |  |  |
|  | Bacillus kochii | 1 |  |  |  |  |  |  |  |
|  | Bacillus sp. |  |  | 1 |  |  |  |  |  |
|  | Staphylococcus sp. |  |  | 1 |  |  |  |  |  |
|  | Staphylococcus warneri | 1 |  |  |  |  |  |  |  |
| Proteobacteria | Acinetobacter junii | 1 |  |  |  |  |  |  |  |
|  | Agrobacterium tumefaciens | 3 |  |  |  |  |  |  |  |
|  | Asaia bogorensis | 1 |  | 2 |  |  |  | 1 | 1 |
|  | Asaia krungthepensis |  |  | 1 |  |  |  | 1 |  |
|  | Asaia siamensis | 1 |  | 1 |  |  |  |  |  |
|  | Asaia spathodeae |  |  | 1 |  |  |  |  | 1 |
|  | Brachybacterium sp. | 1 |  |  |  |  |  |  |  |
|  | Brevibacterium sp. | 1 |  |  |  |  |  |  |  |
|  | Enterobacter sp. | 2 |  |  |  |  |  |  |  |
|  | Haematobacter massiliensis | 1 |  |  |  |  |  |  |  |
|  | Klebsiella pneumoniae subsp. pneumoniae | 1 |  |  |  |  |  |  |  |
|  | Knoellia sinensis |  |  | 1 |  |  |  |  |  |
|  | Knoellia sp. | 1 |  |  |  |  |  |  |  |
|  | Moraxella sp. |  |  | 1 |  |  |  |  |  |
|  | Pantoea agglomerans | 1 |  |  |  |  |  |  |  |
|  | Pantoea stewartii subsp. stewartii | 1 |  |  |  |  |  |  |  |
|  | Rhizobium radiobacter | 1 |  |  |  |  |  |  |  |
|  | Rhizobium sp. | 1 |  |  |  |  |  |  |  |
|  | Rhodococcus equi | 1 |  |  |  |  |  |  |  |
|  | Stenotrophomonas sp. | 2 |  |  |  |  |  |  |  |
|  | Xenophilus aerolatus | 1 |  |  |  |  |  |  |  |
|  | Xenophilus sp. | 1 |  |  |  |  |  |  |  |
