# Supplementary material for: Diversity of the Bacterial Microbiota of Anopheles Mosquitoes from Binh Phuoc Province, Vietnam
Source: Front Microbiol. 2016 Dec 23;7:2095. doi: 10.3389/fmicb.2016.02095 (PMC5181100; doi:10.3389/fmicb.2016.02095)
Supplement: Supplementary file 2 [file Data_Sheet_1.DOCX]

**Supplementary Material**

**Diversity of the bacterial microbiota of Anopheles mosquitoes from Binh Phuoc Province, Vietnam**

**Chung Thuy Ngo, Sara Romano-Bertrand*, Sylvie Manguin and Estelle Jumas-Bilak**

*** Correspondence:** Sara Romano-Bertrand: sara.romano-bertrand@univ-montp1.fr

**Supplementary Fasta File.** Sequences obtained from the 393 TTGE bands

Supplement fasta file_sequences obtained from TTGE bands

>9_HDA1F_68 COL13-08IV

CGCCGTACGTCGAGG

CGCACGGACGAGTAGAACTTGAGGGCGCGACCACCTGTCGTTGTCTCCGG

GGAGCCAAACATGACACCGATCTTCTCGCGCAGCTGATTAATGAATATGG

>201_HDA-1F_42 COL13-06GQ

AATTCGCATGGACGAAGTCTGACGGAGCACGCCGCGTGAGTGATGAAG

GCTTTCGGGTCGTAAAACTCTGTTGTTAGGGAAGAACAAGTGCTAGTTGA

ATAAGCTGGCACCTTGACGGTACCTAACCAGAAAGCCACGGCTAACTACG

TGCCAGCAGCCGCGGTAATA

>202_HDA-1F_43 COL13-06GQ

GATTCGCATGGACGAAGTCTGACGGAGCAACGCCGCGTGAGTGATGAAG

GCTTTCGGGTCGTAAAACTCTGTTGTTAGGGAAGAACAAGTGCTAGTTGA

ATAAGCTGGCACCTTGACGGTACCTAACCAGAAAGCCACGGCTAACTACG

TGCCAGCAGCCGCGGTAATACA

>203_HDA-1F_44 COL13-06GQ

AATTCGCATGGACGAAGTCTGACGGAGCACGCCGCGTGAGTGATGAA

GGCTTTCGGGTCGTAAAACTCTGTTGTTAGGGAAGAACAAGTGCTAGTTG

AATAAGCTGGCACCTTGACGGTACCTAACCAGAAAGCCACGGCTAACTAC

GTGCCAGCAGCCGCGGTAATA

>204_HDA-1F_45 COL13-06GQ

AATCGCATGGACGAAGTCTGACGGAGCAACGCCGCGTGAGTGATGAA

GGCTTTCGGGTCGTAAAACTCTGTTGTTAGGGAAGAACAAGTGCTAGTTG

AATAAGCTGGCACCTTGACGGTACCTAACCAGAAAGCCACGGCTAACTAC

GTGCCAGCAGCCGCGGTAATACA

>205_HDA-1F_46 COL13-06GQ

ATGGGGGCACCCTGATCCAGCCATGCCGCGTGTGTGAAGAAGG

CCTTTTGGTTGTAAAGCACTTTAAGCAGGGAGGAGAGGCTAATGGTTAAT

ACCCATTAGATTAGACGTTACCTGCAGAATAAGCACCGGCTAACTCTGTG

CCAGCAGCCGCGGTAATACA

>206_HDA-1F_47COL13-06GQ

GGGAGGTAGTGACGAGAAATAACAATATGGACC

TCTCTAACGATGGTCCATAATTGGAATGAGTTGAGCATAAATCCTTCAGC

AAGGATCAAGTGGAGGGCAAGTCTGGTGCCAGCAGCCGCGGTAATAATGC

C

>207_HDA-1F_48 COL13-06GQ

CGGGGAGGTAGTGACGAGAAATAACAATATGGACCT

CTCTAATGATGGTCCATAATTGGAATGAGTTGAGCATAAATCCTTCAGCA

AGGATCAAGTGGAGGGCAAGTCTGGTGCCAGCAGCCGCGGTAATACTGCC

CTA

>208_HDA-1F_49 COL13-06GQ

GAGAAGAATATATGACCGTATCT

GAGGAATAAGCGTCGGCTAACTCTGTGCCAGCAGCCCCGGTAAT

>209_HDA-1F_50 COL13-06GQ

GGCACGGGGAGGTAGTGACGAGAAATAACAATATGGACCT

CTCTAATGATGGTCCATAATTGGAATGAGTTGAGTATAAATCCTTCAGCA

AGGATCAAGTGGAGGGCAAGTCTGGTGCCAGCAGCCGCGGTAATACAG

>210_HDA-1F_51 COL13-06GQ

GCACGGGGAGGTAGTGACGAGAAATAACAATATGGACC

TCTCTAATGATGGTCCATAATTGGAATGAGTTGAGTATAAATCCTTCAGC

AAGGATCAAGTGGAGGGCAAGTCTGGTGCCAGCAGCCGCGGTAATACT

>211_HDA-1F_52 COL13-06GQ

AATATGGA

CCTCTCTAACGATGGTCCATAATTGGAATGAGTTGAGCATAAATCCTTCA

GCAAGGATCAAGTGGAGGGCAAGTCTGGTGCCAGCAGCCGCGGTAATACA

GC

>212_HDA-1F_53 COL13-06GQ

CACGGGGAGGTAGTGACGAGAAATAACAATATGGACCT

CTCTAACGATGGTCCATAATTGGAATGAGTTGAGCATAAATCCTTCAGCA

AGGATCAAGTGGAGGGCAAGTCTGGTGCCAGCAGCCGCGGTAATAATG

>213_HDA-1F_54 COL13-06GQ

GCACGGGGAGGTAGTGACGAGAAATAACAATATGGACCT

CTCTAATGATGGTCCATAATTGGAATGAGTTGAGTATAAATCCTTCAGCA

AGGATCAAGTGGAGGGCAAGTCTGGTGCCAGCAGCCGCGGTAATAATGA

>214_HDA-1F_55 COL13-06GQ

CACGGGGAGGTAGTGACGAGAAATAACAATGTGGACC

TCTCTAATGATGGTCCATAATTGGAATGAGTTGAGTATAAATCCTTCAGC

AAGGATCAAGTGGAGGGCAAGTCTGGTGCCAGCAGCCGCGGTAATACAAT

TT

>247_HDA-1F_56 COL13-06GQ

CGCAGCCTGATCCAGCAATGCCGCGTGCGGATGAAGGCCTTCGGGTTGTAAACCGCTTTTGTACGGGACGAAAAGGCTGGGCCTAGGGG

AC

>248_HDA-1F_57 COL13-06GQ

GGCGCAGCCTGATCCAGCAATGCCGCGTGCAGGATGAAGGCCTTCGGGTTGTAAACTGCTTTTGTACGGAACGAAAAAGCTTCTCCTAAT

ACCAGA

>249_HDA-1F_58 COL13-06GQ

AAGTCTGACGGAGCAACGCCGCGTGAGTGATGAAGGCTTTCGGGTCGTAAAACTCTGTTGTTAGGGAAGAACAAGTGCTAGTTG

AATAAGCTGGCACCTTGACGGTACCTAACCAGAAAGCCACGGCTAAATAC

G

>250_HDA-1F_59 COL13-06GQ

GAATCGCATGGACGAAGTCTGACGGAGCAACGCCGCGTGAGTGATGAAGGCTTTCGGGTCGTAAAACTCTGTTGTTAGGAAGAACAAGTGCTAGTTGAATAAGCTGGCACCTTGACGGTACCTAACCAGAAAGCCACGGCTAACTACGTGCCAGCAGCCGCGGTAATAAT

>251_HDA-1F_60 COL13-06GQ

GATTCGCATGGACGAAGTCTGACGGAGCACGCCGCGTGAGTGATGAAGGCTTTCGGGTCGTAAAACTCTGTTGTTAGGGAAGAACAAGTGCTAGTTGAATAAGCTGGCACCTTGACGGTACCTAACCAGAAAGCCACGGCTAACTACGTGCCAGCAGCCGCGGTAATAA

>252_HDA-1F_61 COL13-06GQ

GAGCCTGACGGAGCACGCCGCGTGAGTGATGAAGGCC

TTCGGGTCGTAAAACTCTGTTATTAGAGAAGAACAAATGGTA

>253_HDA-1F_62 COL13-06GQ

CAGTCTTGACGGTACCTTATCAAAAGCCCCGGCTAACTACGTGCC

AGCAGCCGCGGTAATACA

>254_HDA-1F_63 COL13-06GQ

GTAACTATGCGCCCCTTGACGGTACCTAATCAAAAAGCCCCGGCTAACTACGTGCCAGCAGCCGCGGTAATACAAATCGCAATCGGG

>255_HDA-1F_64 COL13-06GQ

GAGTCTGATCCAGCCCGCCGCGTGAGTGATGAAGG

CCTTCGGGTTGTAAACTGCTTTT

>257_HDA-1F_66 COL13-06GQ

GCCTCAGTAACTGCTGGGCCCTTGACGGTACCTAACCAAAAAGCCCCGGCTAACTACGTGCCAGCAGCCGCGGTAATACAACTATACCCCG

>258_HDA-1F_67 COL13-06GQ

GCGAGCCTGATCCAGCAATGCCGCGTGAGTGATGAAGGCCTTCGGGTTGTAAAGCTCTTTTGCCAGGGATGAAAATGACAGTACCTG

>259_HDA-1F_68 COL13-06GQ

GCGAGCCTGATCCAGCAATGCCGCGTGAGTGATGAAGGCCTTCGGGTTGTAAAGCTCTTTTGTCAGGGATGAAAATGACAGTACCTGG

AGA

>260_HDA-1F_70 COL13-06GQ

GGGCGAGCCTGATCCAGCAATGCCGCGTGAGTGATGAAGGCCTTCGGGTTGTAAAGCTCTTTTGCCAGGGATGAAAATGACAGTACCTGG

AGAA

>261_HDA-1F_69 COL13-06GQ

GCAGCCTGATCCAGCAATGCCGCGTGAGTGATGAAGGCCTTCGGGTTGTAAAGCTCTTTTGCCAGGGATGAAAATGACAGTACC

TGGAGAA

>262_HDA-1F_71 COL13-06GQ

ATGGGCGAGCCTGATCCAGCATGCCGCGTGAGTGATGAAGGCCTTAGGGTTGTAAAGCTCTTTTAATAGGGAAGATAATGACGGTACCTATA

GAAGAAGCCCCGGCTAACTCCGTGCCAGCAGCCGCGGTAATACTACGTGC

CCGC

>263_HDA-1F_72 COL13-06GQ

GAGCCTGATCCAGCAATACCGCGTGAGTGATGAAGGCCTTAGGGTTGTAAAGCTCTTTTAATAGGGAAGATAATGACGGTACCTA

TAGAAAAAGCCCCGGCTAACTCCGTGCCAGCAGCCGCGGTAATACTGACG

TGCCAGCAGCCGCGGTAATACA

>264_HDA-1F_73 COL13-06GQ

GGGCGAGCCTGATCCAGCAATGCCGCGTGAGTGATGAAGGCCTTCGGGTTGTAAAGCTCTTTTGCGGGGAAGATAATGACGGTACCC

ATAGAAGAAGC

>265_HDA-1F_74 COL13-06GQ

CCTGGGCGCAGCCTGATCCAGCCATGCCGCGTGCAGTGATGAAG

GCCTTCGGGTTGTAAACCGCTTTTGTACGGAAC

>266_HDA-1F_75 COL13-06GQ

GCCATGGGCGCAGCCTGATCCAGCAATGCCGCGTGCAGTGATGAA

GGCCTTCGGGTTGTAAACCGCTTTTGTACGGAAC

>267_HDA-1F_76 COL13-06GQ

GCTGGGCGCAGCCTGATCCAGCAATGCCGCGTGCAGTGATGAAGGCCTTCGGGTTGTAAACTGCTTTTGTACGGAACGAAAAGGCTCTGGTT

AA

>268_HDA-1F_77 COL13-06GQ

GCAGCCTGATGCAGCGACGCCGCGTGAGTGATGAAGGCCTTCGGGTTGTAAACCTCTTTTACCAGGGACGAAACGGATTTGGCGGAT

C

>269_HDA-1F_78 COL13-06GQ

GGAGCCTGATGCAGCAACGCCGCGTGAGTGATGAAGGCCTTCGGGTTGTAAACCTCTTTTGTAGGGACGAAATGGCTTCG

>270_HDA-1F_79 COL13-06GQ

CACCGGCTAACTACGTGCCAGCAGCCGCGGTAATACAAGCGGGGGCACGGGGGGGACTCCTACGGGAGGCAACAGTGCCGAATCT

>271_HDA-1F_80 COL13-06GQ

GGAGCCTGATCCAGCAATGCCGCGTGAGTGATGAAGGCCTTCGGGTTGTAAACCTCTTTTGTCGGGGACGAAATGGCTTGG

>272_HDA-1F_81 COL13-06GQ

CGAGCTGATCCAGCATGCCGCGTGAGTGAAGAAGGCCTTCGGGTCGTAAAACTCTGTTATCAGAGAAGAACAAATTGTAGAGTAACTGCTACAGTCTTGACGGTATCTTATCAGAAAGCCACGGCTAACTACGTGCCAGCAGCCGCGGTAATACAAATTCTAAATTTGGGGA

>273_HDA-1F_82 COL13-06GQ

GTAAGTA

ACTGTGCACGTCTTGACGGTACCTAATCAAAAAGCCCCGGCTAACTACGT

GCCAGCAGCCGCGGTAATACA

>274_HDA-1F_83 COL13-06GQ

GGCGAGCCTGATCCAGCATGCCGCGTGCGGATGAAGGCCT

TCGGGTTGTAAACTGCTTTTGTCCGGGACGAAAAAGCTT

>275_HDA-1F_84 COL13-06GQ

GGGCGAGCCTGATCCAGCAATGCCGCGTGCAGGATGAAGGCCTTCGGGTTGTAAACTGCTTTTGTACGGAACGAAAAAGCTTCTCCTAATACGAGAGGCCCATGACGGTACCGTAAGAATAAGCACCGGCTAACTACGTGCCAGCAGCCGCGGTAATACA

>278_HDA-1F_87 COL13-06GQ

GCCTGATGCAGCCATGCCGCGTGTGTGATGAA

GGCCTTATGGTTGTAAAGCCCTTTCGCCGAGGAGGAGGCTACTGA

>279_HDA-1F_88 COL13-06GQ

GAGCCTGATGCAGCCACGCCGCGTGTGTGATGAAGGCCTTTTGGTTGTAAAGCACTTTAAGCGAGGAGGAGGCTACTGGTATT

AATACTCCCAGATACTGGACGTTACTCGCAGAATAAGCACCGGCTAACTC

TGTGCCAGCAGCCGCGGTAATACAC

>280_HDA-1F_89 COL13-06GQ

AACCGAGGAGGAGGGTACTGGTATT

AATACTACCAGGTACTGGACGTTACTCGCAGAATAAGCACCGGCTAACTC

TGAG

>281_HDA-1F_90 COL13-06GQ

ACCTGATGCAGCCATGCCGCGTGTGTGATAGACGGCCTTATGGTTGTAAAGCACTTTAAGCGAGGAGGAGGGTACTGGTATTAA

TACTACCAGGTACTGGACGTTACTCGCAGAATAAGCACCGGCTAACTCTG

TGCCAGCAGCCGCGGTAATACA

>282_HDA-1F_91 COL13-06GQ

GCGGACCTGATCAGCCATGCCGCGTGTGTGAGAAGGCCTTATGGTTGTAAAGCACTTTAAGCGAGGAGGAGGGTACTGGTATTAATA

CTACCAGGTACTGGACGTTACTCGCAAAATAAGCACCGGCTAACTCTGTG

CCAGCAGCCGCGGTAATACA

>284_HDA1F_2 COL13-088X

CCAGCACTGCCGCGTGCGGGATG

AAGGCTCTTCGGGTTGTAAACTGCTTTTGTT

>285_HDA1F_3 COL13-088X

GACTGCAGCAACTGCCGCGTGACGGG

ATGAAGGCTCTTCGGGTTGTAAACTGCTTTTGTTCGGGACGAACAGTCTT

GG

>286_HDA1F_4 COL13-088X

GTCCTGATGCAGCAATGCCGCGTGAGGGAT

GACGGCCTTCGGGTTGTAAACCGCTTTTGTCCGGGAAGAAGCGCGTG

>287_HDA1F_5 COL13-088X

CACGAAGTCTGACGGAGCAACGCCGCGTGAGTGATGAAGGCTTTCGGGTCGTAAAACTCTGTTGTTAGGGAAGAACAAGTGCTAGTTGAATAAGCTGGCACCTTGACGGTACCTAACCAGAAAGCCACGGCTAACTACGTGCCAGCAGCCGCGGTAATACA

>288_HDA1F_6 COL13-088X

GTCTGCTCCAGCAATGCCGCGTGAGGGATG

AAGGCCTTCGGGTTGTAAACTGCTTTTGTTCGGAACG

>289_HDA1F_7 COL13-088X

GTCTGACTCCAGCAATGCCGCGTGAGTG

ATGAAGGTCCTTTCGGAGTTCGTAAGCTCTGTTGTTAGGGAAGAACAAGT

GCTGTTTGAATAGGGTGGCACCTTGACGGTACCTAACCAGAAAGCCACGG

CTAACTACGTGCCAGCAGCCGCGGTAATACA

>290_HDA1F_8 COL13-088X

GGCGAAGCCTGATCCAGCAATGCCGCGTGTGTGAAGAAGGTCTTCGGATTGTAAAGCACTTTCGACGGGGACGATGATGACGGTACC

CGTAGAAGAAGCCCCGGCTAACTTCGTGCCAGCAGCCGCGGTAATACA

>291_HDA1F_9 COL13-088X

TGGGCGAGCCTGATCCAGCAATGCCGCGTGTGTGAAGAAGGTCTTCGGATTGTAAAGCACTTTCGACGGGGACGATGATGACGGTACCCGTA

GAAGAAGCCCCGGCTAACTTCGTGCCAGCAGCCGCGGTAATACA

>292_HDA1F_10 COL13-088X

ACGAAGTCTGACGGAGCACGCCGCGTGAGCGAAGAAGGTCTTCGGATTGTAAAGCTCTGTTGTTAGGGAAGAACAAGTACCGTTCG

AATAGGGCGGTACCGTGACGGTACCTAACGAGAAAGCCACGGCTAACTCC

G

>294_HDA1F_12 COL13-088X

GAAGTCTGACGGAGCACGCCGCGTGAGCGATGAAG

GTCTTCGGATTGTAAGTTCTG

>295_HDA1F_13 COL13-088X

ACGAAGTCTGACGGAGCACGCCGCGTGAGCGAAGAAGGTCTTCGGATTGTAAAGCTCTGTTGTTAGGGAAGAACAAGTACCGTTCG

AATAGGGCGGTACCGTGACGGTACCTAACGAGAAAGCCACGGCTAACTAC

GTGCCAG

>296_HDA1F_14 COL13-088X

ACGAAGTCTGACGGAGCACGCCGCGTGAGCGATGAAGGCCTTCGGGTCGTAAAGCTCTGTTGTTAGGGAAGAACAAGTAACGCACTA

ACTGGCG

>297_HDA1F_15 COL13-088X

GGGCGAGCCTGATCCAGCAATGCCGCGTGCAGGATGAAGGCCCTCGGGTTGTAAACTGCTTTTGTACGGAACGAAAAGCCTGGGGCTAATATCCCCGGGTCATGACGGTACCGTAAGAATAAGCACCGGCTAACTACGTGCCAGCAGCCGCGGTAATACA

>298_HDA1F_16 COL13-088X

CGCAGCCTGATCCAGCATGCCGCGTGTGTGAAGAAGGCCTTCGGGTTGTAAAGCACTTTAATTTGGGAAGAAAAAACTCGCTCTAA

TCTGTG

>299_HDA1F_17 COL13-088X

GCGCAGCCTGATCAGCATGCCGCGTGAGGAGAAGCCTTCGGGTTGTAAAGCTCTTTTTGTTGGGAAGAAAAACTACCCTCTAAAGGG

A

>300_HDA1F_18 COL13-088X

CCTGCCGCGTGTGTAAGAAGCCT

TCTGGTTGTAAAGCACTTTTGGCGAGGAGGAGGCTCTTCTAGTTAATACC

TAGGATGA

>301_HDA1F_19 COL13-088X

CAGTCTGATCGAGCATGCCGCGTGAGGGAAGAAGG

CCTTCGGGTCGTGAACTTCTTTTCCCG

>302_HDA1F_20 COL13-088X

CGCAGTCCTGATCCAGCAATGCCGCGTGTGTGAAGAAGGCCTTCGGGTTGTAAAGCTCTTTAATTTGGGAAGAAAAAACTCCCTCT

AACACTGTGAGTCTTGACGGTACCCACAGAATAAGC

>303_HDA1F_21 COL13-088X

GAGCGAGCCTGATCCAGCAATGCCGCGTGCAGGATGAAGGCCTTCGGGTTGTAAACTGCTTTTGTACGGAACGAAAAAGCTTCTCCTAATACGAGAGGCCCATGACGGTACCGTAAGAATAAGCACCGGCTAACTACGTGCCAGCAGCCGCGGTAATACAA

>304_HDA1F_22 COL13-088X

GTACC

GTTCGATTAGGGCGGTCCTGTGACGGTCCCTAAGAAGAAAGCCCCGGTTA

ACTACGTGCCA

>305_HDA1F_23 COL13-088X

ACGAGTCTGACGGAGCACTGCCGCGTGAGAGGAAG

AAGGCCTTCGGGTCGTGAGTTCT

>306_HDA1F_24 COL13-088X

CGAGCCTGATCCAGCATGCCGCGTGCGGATGAAGGCCCTCGGGTTGTAAACTGCTTTTGTACGGAACGAAAAGCCTGGGGCTAAT

ATCCCCGGGTCATGACCGTACCCTAAG

>307_HDA1F_25 COL13-088X

AGCCTGACGCGAGCAATGCCGCGTGAAGGTGAAGAAGGCTCTTCCGGGTCTGTAAACTCTCTTTTCTCGGAGAAGAATAAATGA

CGGTATCTGAGGAATAAGCATCGGCTAACTCTGTGCCAGCAGCCGCGGTA

ATACCGT

>308_HDA1F_26 COL13-088X

GGGCGAGCCTGATCCAGCATGCCGCGTGAGTGAAGAAGGCCTTCGGGTTGTAAAGCTCTTTTGTCAGGGAAGAAACGGTGAGAGCTAATATCTCTTGCTAATGACGGTACCTGAAGAATAAGCACCGGCTAACTACGTGCCAGCAGCCGCGGTAATACAA

>309_HDA1F_27 COL13-088X

CGAGTCTGATCGAGCATGCCGCGTGAGTGAAGAAGGCCTTCGGGTTGTAAAGCTCTTTTGTCAGGGAAGAACCAGTGAGAGCTAGTA

TCTCTTGCTAATGACGGTACCTGAAGAATAAGCGCCGGCTAACTACGTGC

CAGCAGCCGCGGTAATACAA

>310_HDA1F_28 COL13-088X

CTGATCCAGCAATGCCGCGTGAGTGATGAA

GGTCTTCGGATTGTAAACCTCTTTTGCCAGGGATG

>311_HDA1F_29 COL13-088X

CGAGCCTGATCCAGCAATGCCGCGTGAGTGATGAAGGCCTTAGGGTTGTAAAGCTCTTTTACCCGGGATGATAATGACAGTACCGGG

AGAATAAGCTCCGGCTAACTCCGTGCCAGCAGCCGCGGTAATAC

>312_HDA1F_30 COL13-088X

GCGCAGCCTGATCCAGCAATGCCGCGTGAGTGATGAAGGCCTTAGGATTGTAAAGCTCTTTCACCCGGGATGATAATGACAGTACCGG

GAGAATAAGCTCCGGCTAACTCCGTGCCAGCAGCCGCGGTAATACA

>313_HDA1F_31 COL13-088X

GGAGCCTGATCAGCCATGCCGCGTGTGTGATAGAAGGCCTTATGGTTGTAAAGCACTTTAAGCGAGGAGGAGGCTACTTTAGTTA

ATACCTAGAGATAGTGGACGTTACTCGCAGAATAAGCACCGGCTAACTCT

GTGCCAGCAGCCGCGGTAATACA

>314_HDA1F_32 COL13-088X

CGGAGCCTGATCCAGCCATGCCGCGTGCGGGATGAAGGCCTTCGGGTTGTAAACCGCTTTTGTCCGGAACGAAACGGTCTTTTCTA

ATACAGAAGGCTCATGACCGTACCGTTA

>315_HDA1F_33 COL13-088X

GAGCCTGATCAGCCATGCCGCGTGTGTGATAGAAGGCCTTATGGTTGTAAAGCACTTTAAGCGAGGAGGAAGCTCTTCTAACTAAT

ACCTAAGATGAGTGGACGTTACTCGCAGAATAAGCACCGGCTAACTCTGT

GCCAGCAGCCGCGGTAATACA

>316_HDA1F_34 COL13-088X

GAAGCCTGATCCAGCCATGCCGCGTGAGGGATGACTGC

CTTCTGGTTGTAAACCTCTTTCTGCTGGGACAAAAA

>317_HDA1F_35 COL13-088X

CGAAGCCTGATGCAGCACGCCGCGTGAGGGATGACGGCCTTCGGGTTGTAAACCTCTTTCAGCACGGACGAAGCGAAAGTGACGGTAC

CTGCAGAAGAAGGACCGGCCAACTACGTGCCAGCAGCCGCGGTAATACA

>318_HDA1F_36 COL13-088X

GGAGCCTGATCAGCCATGCCGCGTGTGTGAAGAAGGCCTTATGGTTGTAAAGCACTTTAAGCGAGGAGGAGGCTCTTCTAGTTAAT

ACCTAAGATGAGTGGACGTTACTCGCAAAATAAGCACCGGCTAACTCTGT

GCCAGCAGCCGCGGTAATACA

>319_HDA1F_37 COL13-088X

GGGCGAGCCTGATCCAGCAATGCCGCGTGTGTGAAGAAGGTCTTCGGATTGTAAAGCACTTTCGACGGGGACGATGATGACGGTACCCG

TAGAAGAAGCCCCGGCTAACTTCGTGCCAGCAGCCGCGGTAATACA

>320_HDA1F_38 COL13-088X

TGAGTGATGAC

GGCCTTAGGGTTGTAAAGCTCTTTTGTCCGGGACGATAA

>321_HDA1F_39 COL13-088X

TGTTAGGGAAGAACAAGTACCGTTCG

AATAGGGCGGTACTGTGACGGTACCTAACGAGAAAGCCACGGCTAAATAC

GTGCCAGCAGCCGCGGTAATACA

>322_HDA1F_40 COL13-088X

GCGCAGCCTGATCCAGCCATGCCGCGTGAGTGATGAAGGCCTTAGGGTTGTAAAGCTCTTTTGTCCGGGACGATAATGACGGTACCGG

AAGAATAAGCCCCGGCTAACTTCGTGCCAGCAGCCGCGGTAATACAATCA

CC

>323_HDA1F_41 COL13-088X

CCTGATCAGCCATGCCGCGTGTGTGAAGAAGGCCTTTTGGTTGTAAAGCACTTTAAGCGAGGAGGAGGCTACTGGTATTAA

TACTACCGGATAGTGGACGTTACTCGCAGAATAAGCACCGGCTAACTCTG

TGCCAGCAGCCGCGGTAATACA

>324_HDA1F_42 COL13-088X

CATGTGCGAGCCTGATGCAGCGACGCCGCGTGAGGGATGACGGCCTTCGGGTTGTAAACCTCTTTCAGCAGGGAAGAAGCGAAAGTGACGGTACCTGCAGAAGAAGCACCGGCTAACTACGTGCCAGCAGCCGCGGTAATACGTGCCAGCAGCCGCGGTAATACA

>325_HDA1F_43 COL13-088X

TTCGCATGGACGAAGTCTGACGGAGCAACGCCGCGTGAGCGAAGAAGGTCTTCGGATTGTAAAGCTCTGTTGTTAGGGAAGAACAAGTACCGTTCGAATAGGGCGGTACCGTGACGGTACCTAACGAGAAAGCCACGGCTAACTACGTGCCAGCAGCCGCGGTAATACA

>326_HDA1F_44 COL13-088X

GCATGTGCGAGCCTGATGCAGCGACGCCGCGTGAGGGATGACGGCCTTCGGGTTGTAAACCTCTTTCAGCAGGGAAGAAGCGAAAGTGACGGTACCT

GCAGAAGAAGCACCGGCTAACTACGTGCCAGCAGCCGCGGTAATACA

>327_HDA1F_45 COL13-088X

GGGCGAGCCTGATCCAGCAATGCCGCGTGTGTGAAGAAGGTCTTCGGATTGTAAAGCACTTTCGACGGGGACGATGATGACGGTACCCGT

AGAAGAAGCCCCGGCTAACTTCGTGCCAGCAGCCGCGGTAATACAACCTC

>328_HDA1F_46 COL13-088X

GGCGAGCCTGATCCAGCAATGCCGCGTGTGTGAAGAAGGTCTTCGGATTGTAAAGCACTTTCGACGGGGACGATGATGACGGTACCCG

TAGAAGAAGCCCCGGCTAACTTCGTGCCAGCAGCCGCGGTAATACA

>329_HDA1F_47 COL13-088X

TCGCATGGACGAAGTCTGACGGAGCAACGCCGCGTGAGTGAAGAAGGTTTTCGGATCGTAAAGCTCTTTCGTTAGGGAGAACGTGCTCTTTGTGAATAATGAGGAGTAATGACGGTACCTAAATAAGAAGCCACGGCTAACTACGTGCCAGCAGCCGCGGTAATACA

>331_HDA1F_49 COL13-088X

TGCATGGGCGGAGCCTGATGCAGCGACGCCGCGTGGGGGATGACGGCCTTCGGGTTGTAAACCCCTTTCGGCAGGGAACAAGCCTTCGGGTGAGGGT

ACCTGCGGAAGAAGCGCCGGCTAACTACGTGCCAGCAGCCGCGGTAATAC

A

>332_HDA1F_50 COL13-088X

CGAAGCCTGATCCAGCCTGCCGCGTGCGTGATGAAGGCCTTCGGGTTGTAAACCGCTTTTGTCCGGGACGAAGATGCTTCTCCTAA

TACTATAGGCCCGTGAC

>333_HDA1F_51 COL13-088X

GGGGGACCTGATCAGCCATGCCGCGTGTGTGAAGAAGGCCTTATGGTTGTAAAGCACTTTAAGCGAGGAGGAGGCTCTTCTAGTTAATACCTAGGATGAGTGGACGTTACTCGCAGAATAAGCACCGGCTAACTCTGTGCCAGCAGCCGCGGTAATACA

>334_HDA1F_52 COL13-088X

GCATGCACGAAGTCTGACGGAGCACGCCGCGTGAGCGAAGAAGGTCTTCGGATTGTAAAGCTCTGTTGTTAGGGAAGAACAAGTACCGTTCGAATAGGGCGGTACCGTGACGGTACCTAACGAGAAAGCCACGGCTAACTACGTGCCAGCAGCCGCGGTAATACA

>335_HDA1F_53 COL13-088X

AGCGGAGCTGATGCAGCACGCCGCGTGCGGGATGACGGCCTTCGGGTTGTAAACCGCTTTCGCCTGTGACGAAGCGTGAG

>336_HDA1F_54 COL13-088X

GCGAAGCCTGATCCAGCAATGCCGCGTGCAGGATGAAGGCCTTCGGGTTGTAAACTGCTTTTGTACGGAACGAAAAAGCTTCTCCTAATACGAGAGGCCCATGACGGTACCGTAAGAATAAGCACCGGCTAACTACGTGCCAGCAGCCGCGGTAATACA

>337_HDA1F_55 COL13-088X

CGCATGGACGAAGTCTGACGGAGCACGCCGCGTGAGCGAAGAAGGCCTTCGGGTCGTAAAGCTCTGTTGTTAGGGAAGAACAAGTAACGCAGTAACTGGCGTTACCTTGACGGTACCTAACGAGAAAGCCACGGCTAACTACGTGCCAGCAGCCGCGGTAATACA

>339_HDA1F_57 COL13-088X

GGCGAGCCTGATGCAGCACGCCGCGTGAGTGATGAAGGTCTTCGGATCGTAAAACTCTGTTATTAGGGAAGAACAAATGTGTAAGTAA

CTATGCACGTATTGACGGTACCTAATCAGAAAGCCACGGCTAACAACGTG

CCAGCAGCCGCGGTAATACA

>340_HDA1F_58 COL13-088X

AGCGAGCCTGATGGAGCAACGCCGCGTGAGTGATGAAGGTCTTCGGATCGTAAAACTCTGTTATTAGGGAAGAACAAATGTGTAAGTAACTATGCACGTCTTGACGGTACCTAATCAGAAAGCCACGGCTAACAACGTGCCAGCAGCCGCGGTAATACA

>341_HDA1F_59 COL13-088X

TGCATGCGCGAGCCTGATGCAGCACGCCGCGTGAGTGATGACGGCCTTCGGGTCGTAAGCTCTGTCCTCGGGACGATATGAGTGT

>342_HDA1F_60 COL13-088X

CGCAGCCTGATGCAGCCCGCCGCGTGAGTGATGACG

GCCTTCGGGTCGTAAAGCTCTGTCTTCATGGGAAATAAT

>343_HDA1F_61 COL13-088X

ATGCGCGAGCCTGATGCAGCAACGCCGCGTGAGCGATGAAGGC

CTTCGGGTTGTAAAGCTCTGTCCTTGGGGAC

>344_HDA1F_62 COL13-088X

GGGCGAGCCTGATGCAGCAACGCCGCGTGAGCGATGAAGGCCTTCGGGTCGTAAAGCTCTGTCCTCAAGGAAGATAATGACGGTACTT

GAGGAGGAAGCCCCGGCTAACTACGTGCCAGCAGCCGCGGTAATACA

>345_HDA1F_63 COL13-088X

GGTACCCAAGG

AGGAAGCCACGGCTAACTACGTGCCAGCAGCCGCGGTAATACA

>346_HDA1F_64 COL13-088X

GCACCCTGATGCAGCCATGCCGCGTGTATGAAGAAGGCCTTAGGGTTGTAAAGTACTTTTGTTAGGGAAGAAAAGTCCGCTGTGAATAATGGCTGATACTGACGGTACCTAAAGAATAAGCACCGGCTAACTCGTGCCAGCAGCCGCGGTAATACACG

>347_HDA1F_65 COL13-088X

GAGGGACCCTGATGCAGCGACGCCGCGTGAACGAAGAAGGTATTCGTATCGTAAAGTTCTGTCCTATGGGAAGATAATGACAGTACCA

TAGAAGAAAGCTCCGGCTAAATACGTGCCAGCAGCCGCGGTAATACA

>348_HDA1F_66 COL13-088X

ATGGGTGCAGCCTGACCAGCCATCCCGCGTGAAGGACGACTGCCCTATGGGTTGTAAACTTCTTTTGTATAGGGATAAACCTACCCTCGTGAGGGTAGCTGAAGGTACTATACGAATAAGCACCGGCTAACTCCGTGCCAGCAGCCGCGGTAATACAGGCGC

>349_HDA1F_67 COL13-088X

GGCGAGCTGATGCAGCACGCCGCGTGCGGATGACGGCCT

TCGGGTTGTAACCTCTGTCGT

>350_HDA1F_68 COL13-088X

TGTTGTTAGGGAAGAACAAGTACCGTT

CGAATAGGGCGGTACCGTGACGGTACCTAACGAGAAAGCCACGGCTAAAT

ACG

>351_HDA1F_69 COL13-088X

AACAAGTACCG

GTCGAATAGGGCGGTACCGTGACGGTACCTAACGAGAAAGCCACGGC

>352_HDA1F_70 COL13-088X

ACGCCTGATGGAGCAACGCCGCGTGAGTGATGAAGGTCTTCGGATCGTAAAACTCTGTTATTAGGGAAGAACAAATGTGT

AAGTAACTATGCACGTCTTGACGGTACCTAATCAGAAAGCCACGGCTAAC

AACGTGCCAGCAGCCGCGGTAATAC

>353_HDA1F_71 COL13-088X

GGACGAAGTCTGACGGAGCACGCCGCGTGAGCGAAGAAGGCCTTCGGGTCGTAAAGCTCTGTTGTTAGGGAAGAACAAGTAACGCAC

TAACTGGCGTTACCGTGACGGTACCTAACGAGAAAGCCACGGGTAACTAC

GTGCCAG

>354_HDA1F_72 COL13-088X

ATGGGCGGAGCTGATGCAGCAACGCCGCGTGCGGGATGACGGCCTTCGGGTTGTAAACCGCTTTCGCCTGTGACGAAGCGTGAGTGACGGTAA

TGGGTAAAGAAGCACCGGCTAACTACGTGCCAGCAGCCGCGGTAATACA

>355_HDA1F_73 COL13-088X

AAACTCTGTTGGTATAGAAGAACGTTGGTG

AGAGTGGAAAGCTCATCAAGTGACGGTAACTACCCAGAAAGGGACGGCTA

ACTACGTGCCAGCAGCCGCGGTAATACA

>356_HDA1F_74 COL13-088X

GGACGAAGTCTGACGGAGCACGCCGCGTGAGCGAAGAA

GGTCTTCGGATTGTAAAGCTCTGTTGTTAGGGAAGAACAAGTACCGTTCG

AATAGGGCGGTACCGTGACGGTACCTAACGAGAAAGCCACGGCTAACTAC

GTGCCAGCAGCCGCGGTAATAC

>357_HDA1F_75 COL13-088X

GGACGAAGTCTGACGGAGCACGCCGCGTGAGCGAAGA

AGGCCTTCGGGTCGTAAAGCTCTGTTGTTAGGGAAGAACAAGTAACGCAG

TAACTGGCGTTACCGTGACGGTACCTAACGAGAAAGCCACGGCTAACTAC

GTGCCAGCAGCCGCGGTAATACA

>358_HDA1F_76 COL13-088X

TGCATGGGCGGAGCCTGATGCAGCAACGCCGCGTGCGGGATGACGG

CCTTCGGGTTGTAAACCGCTTTCGCCTGTGACGAAGCGTGAGTGACGGTA

ATGGGTAAAGAAGCACCGGCTAACTACGTGCCAGCAGCCGCGGTAATACA

A

>359_HDA1F_77 COL13-088X

ATGCATGGGCGCAGCCTGATGCAGCCATGCCGCGTGTATGAAGAAG

GCCTTCGGGTTGTAAAGTACTTTCAGCGGGGAGGAAGGCGATGAGGTTAA

TAACCGCATCGATTGACGTTACCCGCAGAAGAAGCACCGGCTAACTCCGT

GCCAGCAGCCGCGGTAATACAAG

>360_HDA1F_78 COL13-088X

CGCGAGCCTGATGCAGCGATGCCGCGTGTGGGATGACGG

TCTTCGGATTGTAAACCTCTTTCAA

>361_HDA1F_79 COL13-088X

GCAGTCTGATGCAGCAACGCCGCGTGAGTGATGAA

GGCCTTCGGGTTGTAAACCTCTGTTGTC

>362_HDA1F_80 COL13-088X

GCGCAGCCTGATGCAGCCATGCCGCGTGAGTGATGA

AGGCCTTAGGGTTGTAAAGCTCTTTCACCGGGGAAGATAATGACGGGATC

CGGAGAAGAAACC

>363_HDA1F_81 COL13-088X

GCATGGGCGCAGCCTGATGCAGCGACGCCGCGTGAGGGATGAC

GGCCTTCGGGTTGTAAACCTCTTTCAGTAGGGAAGAAGCGAAAGTGACGG

TACCTGCAGAAGAAGCACCGGCTAACTACGTGCCAGCAGCCGCGGTAATA

CA

>364_HDA1F_82 COL13-088X

TGCATGGGCGCAGCCTGATGCAGCAACGCCGCGTGAGGGATGAC

GGCCTTCGGGTTGTAAACCTCTTTTAGCAGGGAAGAAGCGAGAGTGACGG

TACCTGCAGAAAAAGCACCGGCTAACTACGTGCCAGCAGCCGCGGTAATA

CA

>365_HDA1F_83 COL13-088X

CATGGGCGAGCCTGATGCAGCGACGCCGCGTGAGGGATGAC

GGCCTTCGGGTTGTAAACCTCTTTCAGCAGGGAAGAAGCGAAAGTGACGG

TACCTGCAGAAGAAGCACCGGCTAACTACGTGCCAGCAGCCGCGGTAATA

CA

>366_HDA1F_84 COL13-088X

CGAGCCTGATGCAGCGACGCCGCGTGAGGGATGACGG

TCTTCGGATTGTAAGCTCTTTTCAA

>367_HDA1F_85 COL13-088X

CTGCATGGGCGCAGCCTGATGCAGCAACGCCGCGTGAGGGATGACG

GCCTTCGGGTTGTAAACCTCTTTTAGCAGGGAAGAAGCGAGAGTGACGGT

ACCTGCAGAAAAAGCACCGGCTAACTACGTGCCAGCAGCCGCGGTAATAC

ACCTCGCCGCGGTGTCAT

>368_HDA1F_86 COL13-088X

GATGCATGGGCGCAGCCTGATGCAGCGACGCCGCGTGAGGGATGAC

GGCCTTCGGGTTGTAAACCTCTTTCAGTAGGGAAGAAGCGAAAGTGACGG

TACCTGCAGAAGAAGCACCGGCTAACTACGTGCCAGCAGCCGCGGTAATA

CA

>369_HDA1F_87 COL13-088X

GGCTGCATGGGCGCAGCCTGATGCAGCAACGCCGCGTGAGGGACGACG

GCCTTCGGGTTGTAAACCTCTTTTAGCAGGGAAGAAGCGAGAGTGACGGT

ACCTGCAGAAAAAGCACCGGCTAACTACGTGCCAGCAGCCGCGGTAATAC

A

>370_HDA1F_88 sCOL13-088X

CATGCATGGGCGCAGCCTGATGCAGCAACGCCGCGTGAGGGACGACG

GCCTTCGGGTTGTAAACCTCTTTTAGCAGGGAAGAAGCGAGAGTGACGGT

ACCTGCAGAAAAAGCACCGGCTAACTACGTGCCAGCAGCCGCGGTAATAC

A

>371_HDA1F_89 COL13-088X

CTTCGCATGGACGAAGTCTGACGGAGCACGCCGCGTGAGTGATGA

AGGCTTTCGGGTCGTAAAACTCTGTTGTTAGGGAAGAACAAGTGCTAGTT

GAATAAGCTGGCACCTTGACGGTACCTAACCAGAAAGCCACGGCTAACTA

CGTGCCAGCAGCCGCGGTAATAC

>372_HDA1F_90 COL13-088X

AAATCGCATGGACGAAGTCTGACGGAGCACGCCGCGTGAGTGATGA

AGGCTTTCGGGTCGTAAAACTCTGTTGTTAGGGAAGAACAAGTGCTAGTT

GAATAAGTTGGCACCTTGACGGTACCTAACCAGAAAGCCACGGCTAACTA

CGTGCCAGCAGCCGCGGTAATAC

>373_HDA1F_91 COL13-088X

GGCTCGCATGGACGAAGTCTGACGGAGCACGCCGCGTGAGTGATGAAG

GCTTTCGGGTCGTAAAACTCTGTTGTTAGGGAAGAACAAGTGCTAGTTGA

ATAAGTTGGCACCTTGACGGTACCTAACCAGAAAGCCACGGCTAACTACG

TGCCAGCAGCCGCGGTAATAC

>374_HDA1F_92 COL13-088X

ACTTCGCATGGACGAAGTCTGACGGAGCACGCCGCGTGAGTGATGAA

GGCTTTCGGGTCGTAAAACTCTGTTGTTAGGGAAGAACAAGTGCTAGTTG

AATAAGCTGGCACCTTGACGGTACCTAACCAGAAAGCCACGGCTAACTAC

GTGCCAGCAGCCGCGGTAATAC

>375_HDA1F_93 COL13-088X

CATGGGCGAGCCTGATGCAGCGACGCCGCGTGAGGGATGACG

GCCTTCGGGTTGTAAACCTCTTTCAGCAGGGAAGAAGCGAAAGTGACGGT

ACCTGCAGAAGAAGCACCGGCTAACTACGTGCCAGCAGCCGCGGTAATAC

T

>376_HDA1F_94 COL13-088X

GCGAGCCTGATCCAGCAATGCCGCGTGTGTGAAGA

AGGTCTTCGGATTGTAAAGCACTTTCGACGGGGACGATGATGACGGTACC

CGTAGAAGAAGCCCCGGCTAACTTCGTGCCAGCAGCCGCGGTAATACAAA

G

>377_HDA1F_95 COL13-088X

GGGCGAGCCTGATCCAGCAATGCCGCGTGTGTGAAGAAGG

TCTTCGGATTGTAAAGCACTTTCGACGGGGACGATGATGACGGTACCCGT

AGAAGAAGCCCCGGCTAACTTCGTGCCAGCAGCCGCGGTAATACA

>378_HDA1F_96 COL13-088X

GGGCGAGCCTGATCCAGCAATGCCGCGTGTGTGAAGAAG

GTCTTCGGATTGTAAAGCACTTTCGACGGGGACGATGATGACGGTACCCG

TAGAAGAAGCCCCGGCTAACTTCGTGCCAGCAGCCGCGGTAATACT

>379_HDA1F_1 COL13-08HL

ATGGGCGCAGCCTGATCCAGCCATGCCGCGTGAGTGATGAAG

GCCTTAGGGTTGTAAAGCTCTTTCACCGGAGAAGATAATGACGGTATCCG

GAGAAGAAGCCCCGGCTAACTTCGTGCCAGCAGCCGCGGTAATACAAGA

>380_HDA1F_2 COL13-08HL

GTGATGCATGGGCGCAGCCTGATGCAGCAACGCCGCGTGAGGGATGACGG

CCTTCGGGTTGTAAACCTCTTTTAGCAGGGAAGAAGCGAGAGTGACGGTA

CCTGCAGAAAAAGCACCGGCTAACTACGTGCCAGCAGCCGCGGTAATACT

>381_HDA1F_3 COL13-08HL

ATGCATGGGCGCAGCCTGATGCAGCAACGCCGCGTGAGGGATGAC

GGCCTTCGGGTTGTAAACCTCTTTTAGCAGGGAAGAAGCGAGAGTGACGG

TACCTGCAGAAAAAGCACCGGCTAACTACGTGCCAGCAGCCGCGGTAATA

CAG

>382_HDA1F_4 COL13-08HL

GATGCATGGGCGCAGCCTGATGCAGCCATGCCGCGTGTATGAAGAAG

GCCTTCGGGTTGTAAAGTACTTTCAGCGGGGAGGAAGGCGATGAGGTTAA

TAACCGCATCGATTGACGTTACCCGCAGAAGAAGCACCGGCTAACTCCGT

GCCAGCAGCCGCGGTAATACACGCG

>383_HDA1F_5 COL13-08HL

GATGCATGGGCGCAGCCTGATGCAGCCATGCCGCGTGTATGAAGAAG

GCCTTCGGGTTGTAAAGTACTTTCAGCGGGGAGGAAGGCGATGAGGTTAA

TAACCGCATCGATTGACGTTACCCGCAGAAGAAGCACCGGCTAACTCCGT

GCCAGCAGCCGCGGTAATACACGCG

>384_HDA1F_6 COL13-08HL

GGGATGCATGGGCGCAGCCTGATGCAGCCATGCCGCGTGTATGAAGAA

GGCCTTCGGGTTGTAAAGTACTTTCAGCGGGGAGGAAGGCGATGGAGTTA

ATAACTTCATCGATTGACGTTACCCGCAGAAGAAGCACCGGCTAACTCCG

TGCCAGCAGCCGCGGTAATACACGCG

>385_HDA1F_7 COL13-08HL

GATGCATGGGCGCAGCCTGATGCAGCCATGCCGCGTGTATGAAGAAG

GCCTTCGGGTTGTAAAGTACTTTCAGCGGGGAGGAAGGCGATGAGGTTAA

TAACCGCATCGATTGACGTTACCCGCAGAAGAAGCACCGGCTAACTCCGT

GCCAGCAGCCGCGGTAATACACGCG

>386_HDA1F_8 COL13-08HL

ATGCATGGGCGCAGCCTGATGCAGCCATGCCGCGTGTATGAAGAA

GGCCTTCGGGTTGTAAAGTACTTTCAGCGGGGAGGAAGGCGATGAGGTTA

ATAACCGCATCGATTGACGTTACCCGCAGAAGAAGCACCGGCTAACTCCG

TGCCAGCAGCCGCGGTAATACA

>387_HDA1F_9 COL13-08HL

TGCATGGGCGAGCCTGATGCAGCGACGCCGCGTGAGGGATGACG

GCCTTCGGGTTGTAAACCTCTTTCAGCAGGGAAGAAGCGAGAGTGACGGT

ACCTGCAGAAGAAGCGCCGGCTAACTACGTGCCAGCAGCCGCGGTAATAC

AG

>388_HDA1F_10 COL13-08HL

TGCATGGGCGAGCCTGATGCAGCGACGCCGCGTGAGGGATGACGG

CCTTCGGGTTGTAAACCTCTTTCAGCAGGGAAGAAGCGAGAGTGACGGTA

CCTGCAGAAGAAGCGCCGGCTAACTACGTGCCAGCAGCCGCGGTAATACT

>389_HDA1F_11 COL13-08HL

ATGCATGGGCGAGCCTGATGCAGCGACGCCGCGTGAGGGATGACGG

CCTTCGGGTTGTAAACCTCTTTCAGCAGGGAAGAAGCGAGAGTGACGGTA

CCTGCAGAAGAAGCGCCGGCTAACTACGTGCCAGCAGCCGCGGTAATAC

>390_HDA1F_12 COL13-08HL

GCGAGCCTGATCCAGCAATGCCGCGTGTGTGAAGAAGGT

CTTCGGATTGTAAAGCACTTTCGACGGGGACGATGATGACGGTACCCGTA

GAAGAAGCCCCGGCTAACTTCGTGCCAGCAGCCGCGGTAATACAACTAGT

>391_HDA1F_13 COL13-08HL

CGGAGGATGGGCGAGCCTGATCCAGCAATGCC

GCGTGTGTGAAGAAGGTCTTCGGATTGTAAAGCACTTTCGACGGGGACGA

TGATGACGGTACCCGTAGAAGAAGCCCCGGCTAACTTCGTGCCAGCAGCA

AGCAGATAA

>392_HDA1F_14 COL13-08HL

TGGGCGAGCCTGATCCAGCAATGCCGCGTGTGTGAAGAAGGTCTTCGGATTGTAAAGCACTTTCGACGGGGACGATGATGACGGTACCCG

TAGAAGAAGCCCCGGCTAACTTCGTGCCAGCAGCCGCGGTAATACA

>394_HDA1F_16 COL13-08HL

TAATGACGGTACCCGA

GGAGGAAGCCCCGGCTAACTACGTGCCAGCAGCCGCGGTAATACA

>395_HDA1F_17 COL13-08HL

TGCGCGAGCCTGACGGAGCACGCCGCGTGAGTGATGAAGGTCTTCGGATCGTAAAACTCTGTTATTAGGGAAGAACAAATGTGTAAGTAACTATGCACGTCTTGACGGTACCTAATCAGAAAGCCACGGCTAACTACGTGCCAGCAGCCGCGGTAATAC

>396_HDA1F_18 COL13-08HL

GAAGCCGCGGTATTACAACGTGCCAGCAGCCGCGGTAATACAAGAGCAAACGCCTCCACCTCCACCGTGCGTCTGGCCGGCTCCTCCGGTGCCAACCCGTTTGCCTGTATTGCCGCCGGGATCGCCTCCCTGTGGGGACCGGCTCATGGCGGTGCCAACGAAACCTGCCTCAAGATGCTCGAAGAGATTGGCTCTGTGGATCGCATCCCGAGTTCATCGGCCGGGCCAAGGACAAGAACGATCCCTTCCGTCTGATGGGCTTCTGCCACCGGGTTTACAAAAACCACGATCCCCGTGCCCCGGTATGCGCCAAACCTGTCACGAG

GTGCTGAAAGAGTTGCATATCAAGTTTTTGCTGC

>397_HDA1F_19 COL13-08HL

GACGGACTTGA

GGAGGAAGCCCCGGCTAACTACGTGCCAGCAGCCGCGGTAATACAACTGC

C

>398_HDA1F_20 COL13-08HL

ATGACGGTACTTGA

GGAGGAAGCCCCGGCTAACTACGTGCCAGCAGCCGCGGTAATAC

>399_HDA1F_21 COL13-08HL

ATAATGACGGTACTTGAG

GAGGAAGCCCCGGCTAACTACGTGCCAGCAGCCGCGGTAATAC

>400_HDA1F_22 COL13-08HL

GCGAGCCTGATGCAGCAACGCCGCGTGAGTGATGACG

GTCTTCGGATTGTAAAGCTCTGTCTTTGGGGACGATAATG

>401_HDA1F_23 COL13-08HL

ACGGACTC

CGAGGAGGAAGCCCACGGCTAACTACGTGCCAGCAGCCGCGGTAATACA

>402_HDA1F_24 COL13-08HL

GCGCACCCTGATCTAGCCATGCCGCGTGAGTGATGACAGGCCTTACGGGTTGTAAAGCTCTTTCAGCTGGGAAGATAATGACGGTA

CCAGCAGAAGAAGCCCCGGCTAACTCCGTGCCAGCAGCCGCGGTAATACA

ATACA

>403_HDA1F_41 COL13-08HL

GCGAGCCTGATCCAGCATGCCGCGTGCAGGATGAAGGCCTTCGGGTTGTAAACTGCTTTTGTACGGAACGAAAAAGCTTCTCCTAATA

CGAGAGGCCCATGACGGTACCGGAAGAATAAGCACCGGCTAACTACGTGC

CAGCAGCCGCGGTAATACA

>404_HDA1F_42 COL13-08HL

CGATGGGCGAGCCTGACGCAGCCATGCCGCGTGAATGATGAAGGTCTTAGGATTGTAAAATTCTTTCACCGGGGACGATAATGACGGTACCCG

GAGAAAAAGCCCCGGCTAACTTCGTGCCAGCAGCCGCGGTAATAAAACGT

ACCA

>405_HDA1F_43 COL13-08HL

GGGCGAGCCTGATGCAGCCATGCCGCGTGAATGATGAAGG

TCTTCGGATTGTAAAATTCTTTTCCGGGAACG

>406_HDA1F_44 COL13-08HL

ATGGGCGAAGCCTGATGCAGCCATGCCGCGTGCATGATGA

AGGTCTTCGGATTGTAAACTGCTTTTGTACg

>407_HDA1F_45 COL13-08HL

GAGCGAGCCTGACGCAGCCATGCCGCGTGAATGATGAAGGTCTTAGGATTGTAAAATTCTTTCACCGGGGACGATAATGACGGTAC

CCGGAGAAGAAGCCCCGGCTAACTTCGTGCCAGCAGCCGCGGTAATACA

>409_HDA1F_47 COL13-08HL

ATGCATGGGCGCAGCCTGATGCAGCCATGCCGCGTGTATGAAGAAGGCCTTAGGGTTGTAAAGTACTTTCAGCGGGGAGGAAGGTGATAAGGTTAATACCCTTGTCAATTGACGTTACCCGCAGAAGAAGCACCGGCTAACTCCGTGCCAGCAGCCGCGGTAATACACGCG

>410_HDA1F_48 COL13-08HL

GGAGCCTGATGCAGCGACGCCGCGTGAAGGATGACGGCCTTCGGGTTGTAAACCTGCTTTCGCCTGTGACGAAGCGTGAGTGACGG

TAATGGG

>411_HDA1F_49 COL13-08HL

CATGGGCGGAGCCTGATGCAGCAACGCCGCGTGCGGGATGAC

GGCCTTCGGGTTGTAACCGCTTTCGCC

>412_HDA1F_50 COL13-08HL

GGAGCCTGATGCAGCAACGCCGCGTGCGGGATGA

CGGCCTTCGGGTTGTAACTC

>413_HDA1F_51 COL13-08HL

CGAGCCTGATGCAGCGACGCCGCGTGGGGGATGAC

GGCCTTCGGGTTGTAAACCGCTTTCGTCCGGGAAG

>414_HDA1F_52 COL13-08HL

TGCATGGGCGAAGCCTGATGCAGCGACGCCGCGTGGGGGATGACGGCCTTCGGGTTGTAAACCCCTTTCAGCAGGGAAGAAGCGAAAGTGACGGT

ACCTGCAGAAGAAGCACCGGCTAACTACGTGCCAGCAGCCGCGGTAATAC

A

>415_HDA1F_53 COL13-08HL

GTATTAAC

TGGCCGGATAATGACGGTATCCAATCAAAAAGCCCCGGCTAACTACGTGC

CAGCAGCCGCGGTAATACA

>416_HDA1F_54 COL13-08HL

CCTGCCGCGTGTGTGAAGAAGGCCTTATGGTTGTAAACCGCTTTTGTCGAGGAGGAAGCTCTTCTAGTTAATACCTAAGATGAGTGGACGTT

>417_HDA1F_55 COL13-08HL

GGTTGTGGGAGGGGGGGGAGC

>418_HDA1F_56 COL13-08HL

GCGAGCCTGACGCAGCGATGCCGCGTGCGGGATGAAGGCCTTCGGGTTGTAAACCGCTTTCGCCTGGGACGAAATTGGGGGGACCTG

CATG

>419_HDA1F_57 COL13-08HL

CGAGCCTGACGCAGCGATGCCGCGTGCGGGATGAAG

GCCTTCGGGTTGTAAACCGCTTTCAGCTGGGACGAAATTGA

>420_HDA1F_58 COL13-08HL

GGGCGAAGCCTGACGCAGCGATGCCGCGTGCGGGATGAAGGCCTTCGGGTTGTAAACCGCTTTCAGCAGGGACGAAATTGACGGTAC

CTGCAGAAGAAGCCCCGGCCAACTACGTGCCAGCAGCCGCGGTAATACAA

TACA

>421_HDA1F_59 COL13-08HL

ACAGTTCCGATTGCCAGCAGCCGCGGTAATACAGTTCCGATTGCCAGCAGCCGCGGTAATACAGTTCCGATTGCCAGCAGCCGCGGTA

ATACAGTTCCGATTGCCAGCAGCCGCGGTTATAC

>422_HDA1F_60 COL13-08HL

CATGGAGCGCAGCCTGATCTAGCCATGCCGCGTGAGTGATGAAGGCCTTAGGGTTGTAAAGCTCTTTCAGCTGGGAAGATAATGACGGTACCA

GCAGAAGAAGCCCCGGCTAACTCCGTGCCAGCAGCCGCGGTAATACAATC

A

>423_HDA1F_61 COL13-08HL

AGCCGCGGTAATACCAATCATTCTTCCCTAACAACAGAGTTTTACGAACC

GAAATCCTTCTTCACTCACGCGGCGTTGC

>424_HDA1F_62 COL13-08HL

ACACCCCAGCCGCGGCAATACAAATCATTCTTCCCTAACAACAGAGCTTTACGAACCGAAATCCTTCTTCACTCACGCGGCGTTGCTCCGTCAGGCT

>425_HDA1F_63 COL13-08HL

CAGCCTGATCCAGCAATGCCGCGTGAGTGATGAAGGCCTTAGGGTTGTAAAGCTCTTTTACCCGGGATGATAATGACAGTACCGG

GAGAATAAGCTCCGGCTAACTCCGTGCCAGCAGCCGCGGTAATACAATAC

A

>426_HDA1F_64 COL13-08HL

ACGGTACCCG

GAGAAGAAGCCCCGGCTAACTTCGTGCCAGCAGCCGCGGTAATACAATAC

A

>427_HDA1F_65 COL13-08HL

CACCCTGATCTAGCCATGCCGCGTGAGTGATGACGGCCTTAGGGTTGTAAAGCTCTTTCACCTGGGAAGATAATGACGGGACCA

GCA

>428_HDA1F_66 COL13-08HL

ATGGAGCGGAGCCTGATGCAGCAACGCCGCGTGCGGGATGACGGCCTTCGGGTTGTAAACCGCTTTCGCCTGTGACGAAGCGTGAGTGACG

GTAATGGGTAAAGAAGCACCGGCTAACTACGTGCCAGCAGCCGCGGTAAT

ACA

>429_HDA1F_67 COL13-08HL

GGGGACCCTGACACAGCGACGCCGCGTGAGTGAAGAAGGCCTTCGGGTCGTAAAGCTCAATAGTATGGGAAGAAAGAAATGACGGTAC

CATACGAAAGCCCCGGCTAACTACGTGCCAGCAGCCGCGGTAATACAGGC

GT

>430_HDA1F_68 COL13-08HL

CGCGCAGCCTGATGCAGCGACGCCGCGTGGGGGATGACGGCCTTCGGGTTGTAAACTCCTTTCGCTAGGGACGAAGCTTTTGTGACGGTA

CCTAGATAAGAAGCACCGGCTAACTAC

>431_HDA1F_69 COL13-08HL

ATGGGCGCAGCCTGACCGAGCGACGCCGCGTGTGCGATGAAGGCCTTCGGGTTGTAAAGCACTGTCGTGGGGGGGGAAACCTGACCCCCC

CCAGGAGGAAGCACGGGCTAAGTTCGTGCCAGCAGCCGCGGTAATACCAT

TAGC

>432_HDA1F_70 COL13-08HL

T

GCAGCGAGCCTGACGGAGCAAGCCGCGTGAGTGATGAAGGTCTTCGGATCGTAAAACTCTGTTATTAGGGAAGAACAAATGTGTAAGTAACTATGCACGTCTTGACGGTACCTAATCAGAAAGCCACGGCTAACTACGTGCCAGCAGCCGCGGTAATACA

>433_HDA1F_71 COL13-08HL

CAGGGGACCTGATCCAGCCATGCCGCGTGTGTGAAGAAGGCCTTATGGTTGTAAAGCACTTTAAGCGAGGAGGAGGCTACTTTAGTTAATACCTAGAGATAGTGGACGTTACTCGCAGAATAAGCACCGGCTAACTCTGTGCCAGCAGCCGCGGTAATACA

>434_HDA1F_72 COL13-08HL

GAGCCTGACGGAGCATGCCGCGTGGAGGAAGAAGGCCCACGGGTTGTAAACTTCTTTTCCCGGAGAAGAAGCAATGACGGTAT

CTGGGGAATAAGCATCGGCTAACTCTGTGCCAGCAGCCGCGGTAATACAA

T

>435_HDA1F_73 COL13-08HL

TGGAGCGCAGCCTGATGCAGCGACGCCGCGTGGGGGATGACGGCCTTCGGGTTGTAAACTCCTTTCGCTAGGGACGAAGCTTTTGTGACG

GTACCTAGATAAGAAGCACCGGCTAACTACGTGCCAGCAGCCGCGGTAAT

AC

>436_HDA1F_74 COL13-08HL

GGCATGGAGCGCAGCCTGATGCAGCCATGCCGCGTGTATGAAGAAGGCCTTCGGGTTGTAAAGTACTTTCAGCGGGGAGGAAGGGATGGTGCTTAATACGCGCCGTCATTGACGTTACCCGCAGAAGAAGCACCGGCTAACTCCGTGCCAGCAGCCGCGGTAATACACGCGG

>437_HDA1F_75 COL13-08HL

GAAGCCTGATCAGCACATGCCGCGTGCTGTGATAGAAGGCCTTATGGTTGTAAACGCACTTTAAGCGAGGAGGAGGCTCTTCTAGTTAATACCTAGGATGAGTGGACGTTACTCGCAGAATAAGCACCGGCTAACTCTGTGCCAGCAGCCGCGGTAATACA

>438_HDA1F_76 COL13-08HL

TGCATGGAGCGCAGCCTGATGCAGCGACGCCGCGTGAGGGATGACGGCCTTCGGGTTGTAAACCTCTTTCACCAGGGACGAAGCGTAAGTGACGGT

ACCTGGAGAAGAAGCACCGGCCAACTACGTGCCAGCAGCCGCGGTAATAC

A

>439_HDA1F_77 COL13-08HL

GCGGACCTGATCCAGCCATGCCGCGTGTGTGAAGAAGGCCTTATGGTTGTAAAGCACTTTAAGCGAGGAGGAGGCTACTTTGGTTAATACCTAAAGATAGTGGACGTTACTCGCAAAATAAGCACCGGCTAACTCTGTGCCAGCAGCCGCGGTAATACAA

>440_HDA1F_78 COL13-08HL

GAGCGCAGCCTGATCCAGCAATGCCGCGTGCAGGATGAAGGCCTTCGGGTTGTAAACTGCTTTTGTACGGAACGAAAAGCCCTGGGTTAATACCCTGGGGTCATGACGGTACCGTAAGAATAAGCACCGGCTAACTACGTGCCAGCAGCCGCGGTAATACA

>441_HDA1F_79 COL13-08HL

TGATGGAGGGCACCCTGATCTAGCCATGCCGCGTGAGTGATGAAGGCCTTAGGGTTGTAAAGCTCTTTCAGCTGGGAAGATAATGACGGTACCAGCAGAAGAAGCCCCGGCTAACTCCGTGCCAGCAGCCGCGGTAATACTCCGTGCCAGCAGCCGCGGTAATACACGCG

>442_HDA1F_80 COL13-08HL

CATGGAGCGCAGCCTGATGCAGCCATGCCGCGTGTATGAAGAAGGCCTTCGGGTTGTAAAGTACTTTCAGCGGGGAGGAAGGGATGGCGCTTAATACGCGCCGTCATTGACGTTACCCGCAGAAGAAGCACCGGCTAACTCCGTGCCAGCAGCCGCGGTAATACACGCGGCATGGCTGCATCAGGCTTGCGC

CCATTGTGCAATATTCCCCACTGCTGCCTCCCGTAGGAGTA

>443_HDA1F_81 COL13-08HL

TGCGCGCAGCCTGATGCAGCGACGCCGCGTGAGGGATGGAGGCCTTCGGGTTGTAAACCTCTTTCGTCAGGGAAGAAGGGGAAGTGACGG

TACCTGGGGAAGAAGCACCGGCTAAC

>444_HDA1F_82 COL13-08HL

CGGATGAGCGAGCCTGACGGAGCACGCCGCGTGAGTGATGAAGGTCTTCGGATCGTAAAACTCTGTTATTAGGGAAGAACAAATGTGTAAGTAACTATGCACGTCTTGACGGTACCTAATCAGAAAGCCACGGCTAACTACGTGCCAGCAGCCGCGGTAATACA

>445_HDA1F_83 COL13-08HL

GAAGCCTGATGCAGCAATGCCGCGTGCAGGATGAAGGCCTTCGGGTTGTAAACTGCTTTTGTACGGAACGAAAAAGCTTCTCCTAA

TACGAGAGGCCCATGACGGTACCGTAAGAATAAGCACCGGCTAACTACGT

ACCAGCAGCCGCGGTAATACA

>446_HDA1F_84 COL13-08HL

GCGCAGCCTGATGCAGCGACGCCGCGTGAGGGATGACGGCCTTCGGGTTGTAAACCTCTTTCAGTAGGGAAGAAGCGAAAGTGACGGT

ACCTGCAGAAGAAGCACCGGCTAACTACGTGCCAGCAGCCGCGGTAATAC

>447_HDA1F_85

GGCGGAGCCTGATGCAGCAACGCCGCGTGCGGGATGACGGCCTTCGGGTTGTAAACCGCTTTCGCCTGTGACGAAGCGTGAGTGACGGT

AATGGGTAAAGAAGCACCGGCTAACTACGTGCCAGCAGCCGCGGTAATAC

A

>448_HDA1F_86 COL13-08HL

CGCGAGCCTGACGGAGCATGCCGCGTGAAGGAAGAAGGCTCACGGGTCGTAAACTTCTTTTCTCGGAGAAGAATAAATGACGGTAT

CTGAGGAATAAGCATCGGCTAACTCTGTGCCAGCAGCCGCGGTAATACAA

CC

>449_HDA1F_87 COL13-08HL

GTGATGCAGCGACGCCGCGTGAGGGA

TGACGGCCTTCGGGTTGTAAACCGCTTTTGGACTGA

>451_HDA1F_89 COL13-08HL

AGCGCAGCCTGATGCAGCGACGCCGCGTGGGGG

ATGACGGCCTTCGGGTTGTAAACTCCTTTCGCTAGGGAC

>452_HDA1F_90 COL13-08HL

CAGCCTGATCCAGCAATGCCGCGTGCAGGATGAAGGCCCTCGGGTTGTAAACTGCTTTTGTACGGAACGAAAAGCCTGGGGC

TAATATCCCCGGGTCATGACGGTACCGTAAGAATAAGCACCGGCTAACTA

CGTGCCAGCAGCCGCGGTAATACA

>453_HDA1F_91 COL13-08HL

ACCTGATCAGCCATGCCGCGTGTGTGA

AGAAGGCCTTATGGTTGTAAAGCACTTTAAGCGAGGAGGAGGCTCTTCTA

GTTAATACCTAGGATGAGTGGACGTTACTCGCAGAATAAGCACCGGCTAA

CTCTGTGCCAGCAGCCGCGGTAATACA

>454_HDA1F_92 COL13-08HL

CGCAGCCTGATCCAGCATGCCGCGTGCGGTGAT

GAAGGCCTTCGGGTTGTAAACTGCTTTTACCCGGAACGAAAAAGCT

>455_HDA1F_93 COL13-08HL

GCGCAGCCTGATCCAGCAATGCCGCGTGAGTGATAGAAGGCCTTCGGGTTGTAAACCTCTTTTGTCAGGGAAGAAAATGATCTGGC

CT

>456_HDA1F_94 COL13-08HL

GGCGCAGCCTGATGCAGCGACGCCGCGTGGGGGATGACGGCCTTCGGGTTGTAAACTCCTTTCGCTAGGGACGAAGCTTTTT

GTGACGGTACCTAGATAAGAAGCACCGGCTAACTACGTGCCAGCAGCCGC

GGTAATACA

>457_HDA1F_95 COL13-08HL

CTTCGCGCAGCCTGATGCAGCACGCCGCGTGAGGGACGAAGGCCTTCGGGTCGTAAACCTCTTTTAGCAGGGAAGAAGCGAAAGTGACG

GTACCTGCAGAAAAAGCACCGGCTAAC

>459_HDA1F_1 COL13-08IV

CGCAGCCTGATGCAGCAACGCCGCGTGAGGGAC

GAAGGCCTTCGGGTCGTAAACCTCTTTTATCAGGGAAGAAGCGAAA

>460_HDA1F_2 COL13-08IV

TGGAGCGCAGCCTGATGCAGCAACGCCGCGTGAGGGACGAAGGCCTTCGGGTCGTAAACCTCTTTTAGCAGGGAAGAAGCGAAAGTGACG

GTACCTGCAGAAAAAGCACCGGCTAACTACGTGCCAGCAGCCGCGGTAAT

ACA

>461_HDA1F_3 COL13-08IV

CTGGGCGCACCCTGATCCAGCCATGCCGCGTGTGTGAAGAAGGCCTTTTGGTTGTAAAGCACTTTAAGCAGGGAGGAGAGGCTAATGGTTAATACCCATTAGATTAGACGTTACCTGCAGAATAAGCACCGGCTAACTCTGTGCCAGCAGCCGCGGTAATACAA

>462_HDA1F_4 COL13-08IV

GGGCGCAGCCTGATCAGCCATGCCGCGTGCAGGATGAAGGCCTTCGGGTTGTAAACTGCTTTTGTCCGGAACGAAAAAGCTTCT

>463_HDA1F_5 COL13-08IV

CAGCCTGATCCAGCAATGCCGCGTGCAGTGATGAAGGCCTTCGGGTTGTAAACTGCTTTTGTACGGAACGAAAAAGCTTCG

>464_HDA1F_6 COL13-08IV

GCAGCCTGATCAGCATGCCGCGTGCAGGATGAAGGCC

TTCGGGTTGTAAACTGCTTTTGTACGGAACGAAAAAGCTTCG

>464_HDA1F_6 COL13-08IV

GCAGCCTGATCAGCATGCCGCGTGCAGGATGAAGGCC

TTCGGGTTGTAAACTGCTTTTGTACGGAACGAAAAAGCTTCG

>466_HDA1F_8 COL13-08IV

CGCAGCCTGATCCAGCAATGCCGCGTGCAGTGATGAAGGCCTTCGGGTTGTAAACTGCTTTTGTCCGGAACGATAATGCTTC

>465_HDA1F_7 COL13-08IV

CTGATCCAGCCATGCCGCGTGAGTGATGAAG

GCCTTAGGGTTGTAAAGCTCTTTTGCCGGGGACGATAATGACG

>467_HDA1F_9 COL13-08IV

TACC

CGCGATCCATGACGGTACCTGAACAATAAGCCCCGGCTAACTACGTGC

>468_HDA1F_10 COL13-08IV

GAGCCTGATCCAGCATGCCGCGTGCGTGATGAAGGCCCTCGGGTTGTAAACTGCTTTTGTACGGAACGAAAAGCTCGGGGCTAATAC

CCCCGATT

>470_HDA1F_12 COL13-08IV

CGAGCCTGATCCAGCAATGCCGCGTGCAGGATGAAGGCCCTCGGGTTGTAAACTGCTTTTGTACGGAACGAAAAGCTCTGGGCTAATACCCCGGAGTCATGACGGTACCGTAAGAATAAGCACCGGCTAACTACGTGCCAGCAGCCGCGGTAATACAAGTAA

>471_HDA1F_13 COL13-08IV

AAGCCTGATCCAGCCTGCCGCGTGCAGGGATG

AAGGCCCTCGGGTTGTAAACCGCTTTTGTCTGGGA

>472_HDA1F_14 COL13-08IV

GCTGGGCGCAGCCTGATCCAGCATGCCGCGTGAGTGAAGAAGGCCTTCGGGTTGTAAAGCTCTTTTGTCAGGGAAGAAACGGCTGTGGCTAATATCCACGGCTAATGACGGTACCTGAAGAATAAGCACCGGCTAACTACGTGCCAGCAGCCGCGGTAATACA

>473_HDA1F_15 COL13-08IV

GATGGATGGGCGCAGCCTGATCCAGCATGCCGCGTGAGTGAAGAAGGCCTTCGGGTTGTAAAGCTCTTTTGTCAGGGAAGAAACGGCTGTGGCTAATATCCACGGCTAATGACGGTACCTGAAGAATAAGCACCGGCTAACTACGTGCCAGCAGCCGCGGTAATACA

>474_HDA1F_16 COL13-08IV

AGCGCAGCCTGATCCAGCATGCCGCGTGAGTGAAGAAGGCCTTCGGGTTGTAAAGCTCTTTTGTCAGGGAAGAAACGGCTGTGGCTAATATCCACGGCTAATGACGGTACCTGAAGAATAAGCACCGGCTAACTACGTGCCAGCAGCCGCGGTAATACA

>475_HDA1F_17 COL13-08IV

GATGGGCGCAGCCTGATCCAGCATGCCGCGTGAGTGAAGAAGGCCTTCGGGTTGTAAAGCTCTTTTGTCAGGGAAGAAACGGTTGAGGCTAATATCCTTTGCTAATGACGGTACCTGAAGAATAAGCACCGGCTAACTACGTGCCAGCAGCCGCGGTAATACA

>476_HDA1F_18 COL13-08IV

CATGAGCGCAGCCTGATCCAGCATGCCGCGTGAGTGAAGAAGGCCTTCGGGTTGTAAAGCTCTTTTGTCAGGGAAGAAACGGCTGTGGCTAATATCCACGGCTAATGACGGTACCTGAAGAATAAGCACCGGCTAACTACGTGCCAGCAGCCGCGGTAATACA

>477_HDA1F_19 COL13-08IV

GATGGGCGCAGCCTGATCCAGCATGCCGCGTGAGTGAAGAAGGCCTTCGGGTTGTAAAGCTCTTTTGTCAGGGAAGAAACGGCTGTGGCTAATATCCACGGCTAATGACGGTACCTGAAGAATAAGCACCGGCTAACTACGTGCCAGCAGCCGCGGTAATACAA

>478_HDA1F_20 COL13-08IV

TGGGATGGGCGCAGCCTGATCCAGCATGCCGCGTGAGTGAAGAAGGCCTTCGGGTTGTAAAGCTCTTTTGTCAGGGAAGAAACGGCTGTGGCTAATATCCACGGCTAATGACGGTACCTGAAGAATAAGCACCGGCTAACTACGTGCCAGCAGCCGCGGTAATAC

>480_HDA1F_22 COL13-08IV

GCGGAGCCTGATGCAGCACGCCGCGTGCGGGATGACGGCCTTCGGGTTGTAAACCGCTTTCGCCTGTGACGAAGCGTGAGTGACGGTA

ATGGGTAAAGAAGCACCGGCTAACTACGTGCCAGCAGCCGCGGTAATACA

>481_HDA1F_23 COL13-08IV

GCAGCCTGATGCAGCAATGCCGCGTGAGTGATGA

AGGCCTTCGGGTTGTAAAGCTCTTTCGCCTGGGACGATTCGG

>483_HDA1F_25 COL13-08IV

GGATTGTAAAGCTCTTTCACCGGTGAAGATAATGACGGGGCCGGAGAAGAAGCCCCGGCTAACTTCGTGCCAGCAGCCGCGGTAATACAATACAC

GCCCCTCCGCG

>484_HDA1F_26 COL13-08IV

GCCGGAGAAGAAGCCCCGGCTAACTT

CGTGCCAGCAGCCGCGGTAATACAAT

>485_HDA1F_27 COL13-08IV

AGCGCAGCCTGATCCAGCCATGCCGCGTGAGTGATGAAGGTCTTAGGATTGTAAAGCTCTTTCACCGGTGAAGATAATGACGGTAGCC

GGAGAAGAAGCCCCGGCTAACTTCGTGCCAGCAGCCGCGGTAATACA

>488_HDA1F_30 COL13-08IV

GAAGCCTGATCCAGCACGCCGCGTGAGGGATGACGGCCTTCGGGTTGTAAACTGCTTTCGTCCGGAACGAAACAGCTTCGCCTAA

>489_HDA1F_31 COL13-08IV

AGGGACCTGATGCAGCGACGCCGCGTGAACGAAGAAGGTATTCGTATCGTAAAGTTCTGTCCTATGGGAAGATAATGACAGTACCAT

AGAAGAAAGCTCCGGCTAAATACGTGCCAGCAGCCGCGGTAATACA

>490_HDA1F_32 COL13-08IV

TGCAG

GATGAAGGCCTTCGGGTCGTAAACTGCTTTTATGAGTGAAGAATATGACG

GTAACTCATGAAT

>491_HDA1F_33 COL13-08IV

CGAAGCCTGACGGAGCATGCCGCGTGAAGGCAGAAGGCCCACGGGTCATGAACTTCTTTTCTCGGAGAAGAAAAAATGACGG

TATCTGAGGAATAAGCATCGGCTAACTCTGTGCCAGCAGCCGCGGTAATA

CAATA

>493_HDA1F_35 COL13-08IV

CAGTAACTTATAAG

AAAGCTCCGGCTAACTACGTGCCAGCACCCGCGGTAATACAAATACAAGG

GTGGGAGAGA

>494_HDA1F_36 COL13-08IV

GGTGCAGCCTGATCAGCCATCCCGCGTGAAGGACGACTGCCCTATGGGTTGTAAACTTCTTTTGTATAGGGATAAACCTACCCTCGTGAGGGTAGCTGAAGGTACTATACCAATAAGCACCGGCTAACTCCGTGCCAGCAGCCGCGGTAATACAATTCAA

>495_HDA1F_37 COL13-08IV

AGTGCAGCCTGACCAGCCATCCCGCGTGAAGGACGACTGCCCTATGGGTTGTAAACTTCTTTTGTATAGGGATAAACCTACCCTCGTGAGGGTAGCTGAAGGTACTATACCAATAAGCACCGGCTAACTCCGTGCCAGCAGCCGCGGTAATACAATCAA

>496_HDA1F_38 COL13-08IV

GAAGTCTGACGGAGCACGCCGCGTGAGCGATGAAGGCCTTCGGGTCGTAAAGCTCTGTTGTTAGGGAAGAACAAGTACCGTTCG

AATAGGGCGGTACCTTGACGGTACCTAACCAGAAAGCCACGGCTAACTAC

GTGCCAGCAGCCGCGGTAATA

>497_HDA1F_39 COL13-08IV

AGCGCAGCCTGATCCAGCCATGCCGCGTGAGTGATGAAGGCCTTAGGGTTGTAAAGCTCTTTTAGCAGGGAAGATAATGACGGTA

CCTGCAGAAAAAGCCCCGGCTAACTTCGTGCCAGCAGCCGCGGTAATACA

>499_HDA1F_41 COL13-08IV

AAGTCTGACGGAGCACGCCGCGTGAGCGATGAAGGCCTTCGGGTCGTAAAGCTCTGTTGTTAGGGAAGAACAAGTACCGTTC

GAATAGGGCGGTACCTTGACGGTACCTAACCAGAAAGCCACGGCTAACTA

CGTGCCAGCAGCCGCGGTAATAC

>500_HDA1F_42 COL13-08IV

AGGGCACCCTGATCTAGCCATGCCGCGTGAGTGATGAAGGCCTTAGGGTTGTAAAGCTCTTTCAGCTGGGAAGATAATGACGGTA

CCAGCAGAAGAAGCCCCGGCTAACTCCGTGCCAGCAGCCGCGGTAATACA

>501_HDA1F_43 COL13-08IV

AGCCTGATCCAGCATGCCGCGTGAGTGAAGAAGGCCTTCGGATTGTAAAGCTCTTTTGTCAGGGAAGAACCGGTGTGTAGC

TAATA

>502_HDA-1F_1 COL13-0AW5

GAGGGCACCTGACCCAGCGACGCCGCGTGAGGGA

AGACAGCCTTCGGGTTGTAAACCTCTGTTGCAGGGGAAGAAGGACGTGAC

GGTACCCTGCGAGGAAGCTCCGGCTAACTACGTGCCAGCAGCCGCGGTAA

TACATGCCGGGCCGCGGTAATACA

>504_HDA1F_45 COL13-08IV

GCACGCCTGATCCAGCAATGCCGCGTGCAGTGATGAAGGCCCTCGGGTTGTAAACTGCTTTTGTCCGGAACGAAAAGCCT

>506_HDA1F_47 COL13-08IV

GCGGCGCAGCCTGATCCAGCAATGCCGCGTGCAGGAAGAAGGCCTTCGGGTTGTAAACTGCTTTTGTCAGGGAAGAAATCTTCTGGGCTAATACCCCGGGAGGATGACGGTACCTGAAGAATAAGCACCGGCTAACTACGTGCCAGCAGCCGCGGTAATAC

>507_HDA1F_48 COL13-08IV

AGCGAAGCCTGATCCAGCATGCCGCGTGCAGGATGAAGGCCCTCGGGTTGTAAACTGCTTTTGTACGGAACGAAAAGCCTGGGGCTA

ATACCCCCGGGTCATGACGGTACCGTAAGAATAAGCACCGGCTAACTACG

TGCCAGCAGCCGCGGTAATAC

>508_HDA1F_49 COL13-08IV

GCACCTGATGCAGCGACGCCGCGTGAGTGATGAAGCCCTTCGGGGTGTAAAGCTCTTTCGGCCCGGACGATAATGACGGTACGGGA

AGAAGAAGCTGCGGCTAACTACGTGCCAGCAGCCGCGGTAATACAATCA

>509_HDA-1F_2 COL13-0AW5

AAGAAGA

CTTCGGGTTGTAAACCTCTGTTGCAGGGGAAGAAGGAAGTGACGGTACCC

TGTAAGGAAGCTCCGGCTAAC

>510_HDA1F_50 COL13-08IV

GCGCAGCCTGATCCGCATGCCGCGTGAGTGAGAAGGCCTTCGGGTTGTAAAGCTCTTTTGTCAGGGAAGAAACGGCTGTGGCTAAT

ATCCAAGGCTAATGACGGTACCTGAAGAATAAGCACCGGCTAACTACGTG

CCAGCAGCCGCGGTAATACA

>511_HDA1F_51 COL13-08IV

CGCGAAGCCTGATCCAGCATGCCGCGTGAGTGATGAAGGCCTTAGGGTTGTAAAGCTCTTTTACCCGGGATGATAATGACAGTA

CCGGGAGAATAAGCTCCGGCTAACTCCGTGCCAGCAGCCGCGGTAATACA

AATAC

>512_HDA1F_52 COL13-08IV

ATGCAGCGCAGCCTGATCCAGCCATACCGCGTGGGTGAAGAAGGCCTTCGGGTTGTAAAGCCCTTTTGTTGGGAAAGAAATCCAGCTGGCTAATACCCGGTTGGGATGACGGTACCCAAAGAATAAGCACCGGCTAACTTCGTGCCAGCAGCCGCGGTAATAC

>513_HDA1F_53 COL13-08IV

GCTGAGCGAGCCTGATCCAGCATGCCGCGTGCAGGATGAAGGCCCTCGGGTTGTAAACTGCTTTTGTACGGAACGAAAAGCCTGGGGCTAATATCCCCGGGTCATGACGGTACCGTAAGAATAAGCACCGGCTAACTACTGCCAGCAGCCGCGGTAATAC

>514_HDA1F_54 COL13-08IV

CATGGGCGCAGCCTGATCTAGCCATGCCGCGTGGGTGATGAAGGCCTTAGGGTCGTAAAGCCCTTTCGCCGGGGAAGATAATGACGGTAC

CCGGTAAAGAAACCCCGGCTAACTCCGTGCCAGCAGCCGCGGTAATACAA

AAT

>515_HDA1F_55 COL13-08IV

GAAGTCTGACCGAGCACGCCGCGTGAGTGATGA

CGGCCTTCGGGTCGTAAAGCTCTGTTGCCT

>516_HDA1F_56 COL13-08IV

GGGCGGAGCCTGATGCAGCAACGCCGCGTGCGGGATGACGGCCTTCGGGTTGTAAACCGCTTTCGCCTGTGACGAAGCGTGAGTGACGG

TAATGGGTAAAGAAGCACCGGCTAACTACGTGCCAGCAGCCGCGGTAATA

CAAGG

>517_HDA1F_57 COL13-08IV

CATGCAGCGAGCCTGATCCAGCAATGCCGCGTGTGTGAAGAAGGCCTTCGGGTTGTAAAGCACTTTTGTCCGGAAAGAAATCCTTGGCTCTAATACAGTCGGGGGATGACGGTACCGGAAGAATAAGCACCGGCTAACTACGTGCCAGCAGCCGCGGTAATACA

>518_HDA1F_58 COL13-08IV

ATGAGCGCAGCCTGATCCAGCATGCCGCGTGAGTGATGAAGGCCTTAGGGTTGTAAAGCTCTTTTACCAGGGATGATAATGACAGTACCTG

GAGAATAAGCTCCGGCTAACTCCGTGCCAGCAGCCGCGGTAATACA

>519_HDA1F_59 COL13-08IV

GCAGCGAAGCCTGACGGAGCATGCCGCGTGGAGGTAGAAGGCCCACGGGTCGTGAACTTCTTTTCCCGGAGAAGAAGCAATGACGGTA

TCTGGGGAATAAGCATCGGCTAACTCTGTGCCAGCAGCCGCGGTAATACA

AAAGA

>520_HDA-1F_1 COL13-09TV

GCGAAGCCTGATCAGCCTGCCGCGTGCGGATGAAGGCCT

TCGGGTTGTAAACTGCTTTTGTCCGGAACGAAAAGCCTGGGGCTAATATC

CCCGGGTCATGACGGTACCGCAAGAATAAGCACCGGCTAACTACGTGCCA

GCAGCCGCGGTAATACAA

>521_HDA-1F_2 COL13-09TV

TGGGCGAAGCCTGACCCAGCACGCCGCGTGAAGGATGAAGTATCTTCGGTATGTAAACTTCGAAAGAATGGGAAGAATAAATGACGGTACCATTTATAAGCTCCGGCTAACTACGTGCCAGCAGCCGCGGTAATACTACGTGCCAGCAGCCGCGGTAATACAA

>522_HDA-1F_3 COL13-09TV

GGGCGAGCCTGATCCAGCCATGCCGCGTGCAGGATGAAGGCCTTCGGGTTGTAAACTGCTTTTGTACGGAACGAAAAGGTCTTTTCTAATAAAGAAGGCTCATGACGGTACCGTAAGAATAAGCACCGGCTAACTACGTGCCAGCAGCCGCGGTAATAC

>523_HDA-1F_4 COL13-09TV

ATGAGTGCAGCCTGACCAGCCATCCCGCGTGAAGGACGACTGCCCTATGGGTTGTAAACTTCTTTTGTATAGGGATAAACCTACCCTCGTGAGGGTAGCTGAAGGTACTATACGAATAAACACCGGCTAACTCCGTGCCAGCAGCCGCGGTAATACAATACA

>524_HDA-1F_5 COL13-09TV

CGCAGCCTGATCAGCAATGCCGCGTGCAGTGAAGAAGG

CCTTCGGGTTGTAAACTGCTTTTGTTGGGAACGAAATGTT

>525_HDA-1F_6 COL13-09TV

GCGAGCCTGACGCAGCATGCCGCGTGAATGATGAAGGTCTTCGGATTGTAAACTTCTTTCGACTGGGACGATATGGACGGCTCATTT

A

>526_HDA-1F_7 COL13-09TV

CGAGCCTGATCCAGCATGCCGCGTGCAGGATGAAGGCCTTCGGGTTGTAAACTGCTTTTGTACGGAACGAAAAAGCTTCTCCTAATA

CTAGAGGCCCATGACGGTACCGTAAGAATAAGCACCGGCTAACAACGTGC

CAGCAGCCGCGGTAATACA

>527_HDA-1F_8 COL13-09TV

TGGGCGAAGCCTGACGGAGCACGCCGCGTGAGTGATGAAGGTCTTCGGATCGTAAAACTCTGTTATTAGGGAAGAACAAATGTGTAAGTAACTGTGCACGTCTTGACGGTACCTAATCAGAAAGCCACGGCTAACTACGTGCCAGCAGCCGCGGTAATAC

>528_HDA-1F_9 COL13-09TV

GCGCGAGCCTGACGGAGCACGCCGCGTGAGTGATGAAGGTCTTCGGATCGTAAAACTCTGTTATTAGGGAAGAACAAACGTGTAAGTAACTGTGCACGTCTTGACGGTACCTAATCAGAAAGCCACGGCTAACTACGTGCCAGCAGCCGCGGTAATACA

>529_HDA-1F_10 COL13-09TV

TCGCATGGGCGAAGCCTGACGGAGCACGCCGCGTGAGTGATGAAGGTCTTCGGATCGTAAAACTCTGTTATTAGGGAAGAACAAACGTGTAAGTAACTGTGCACGTCTTGACGGTACCTAATCAGAAAGCCACGGCTAACTACGTGCCAGCAGCCGCGGTAATAC

>530_HDA-1F_11 COL13-09TV

CCTGGGCGCAGCCTGATCCAGCCATGCCGCGTGAGTGATGAAGGCCTTAGGGTTGTAAAGCTCTTTCAGTGGGGAAGATAATGACGGTACCCATAGAAGAAGCCCCGGCTAACTTCGTGCCAGCAGCCGCGGTAATACTACGTGCCGCAGCCGCGGTAATACAAAA

>532_HDA-1F_13 COL13-09TV

TCGCATGGGCGAGCCTGACGGAGCATGCCGCGTGAAGGCAGAAGGCCCACGGGTCATGAACTTCTTTTCTCGGAGAAGAAAAAATGACGGTATCTGAGGAATAAGCATCGGCTAACTCTGTGCCAGCAGCCGCGGTAATACCATGCCAGCAGCCGCGGTAATACAA

>533_HDA-1F_14 COL13-09TV

CGCAGTCTGATGCAGCCATGCCGCGTGAATGATGAAGGCCTTCGGGTCGTAAAGTTCTGTTCTTGGGGACGAAAGTGTGGCACCC

C

>534_HDA-1F_15 COL13-09TV

GAGGACTCTGATGCAGCGACGCCGCGTGAACGATGAAGGCCTTCGGGTCGTAAAGTTCTGTCCTTGGGGACGATAATGACGGTACCCAAGGAGGAAGCCCCGGCTAACTACGTGCCAGCAGCCGCGGTAATACAA

>535_HDA-1F_16 COL13-09TV

GGACTCTGATGCAGCGACGCCGCGTGAACGATGAA

GGCCTTCGGGTCGTAAAGTGCTGTCCTTGG

>536_HDA-1F_17 COL13-09TV

GCAGTCTGATGCAGCCATGCCGCGTGTATGATGAAGG

CCTTCGGGTTGTAAAGTACTTTCCTTGGGGACGAAGGGAT

>537_HDA-1F_18 COL13-09TV

CATGGAGACTCTGATGCAGCGACGCCGCGTGAACGATGAAGGCCTTCGGGTCGTAAAGTTCTGTCCTTGGGGACGATAATGACGGTACCCAAGGAGGAAGCCCCGGCTAACTACGTGCCAGCAGCCGCGGTAATACA

>538_HDA-1F_19 COL13-09TV

TCGCATGAGCGAGCCTGACGGAGCACGCCGCGTGAGTGATGAAGGTCTTCGGATCGTAAAACTCTGTTATTAGGGAAGAACAAATGTGTAAGTAACTATGCACGTCTTGACGGTACCTAATCAGAAAGCCACGGCTAACTACGTGCCAGCAGCCGCGGTAATAC

>539_HDA-1F_20 COL13-09TV

CCGTAACTA

TGCACGTCTTGACGGTACCTAATCAAAAAGCCCCGGCTAACTACGTGCCA

GCAGCCGCGGTAATACAA

>541_HDA-1F_21 COL13-09TV

GGAGCGCAGCCTGATGCAGCGACGCCGCGTGGGGGATGACGGCCTTCGGGTTGTAAACTCCTTTCGCTAGGGACGAAGCTTTTTGTGACGG

TACCTA

>542_HDA-1F_22 COL13-09TV

GGAGCGCAGCCTGATGCAGCAACGCCGCGTGAGGGATGACGGCCTTCGGGTTGTAAACCTCTTTTAGCAGGGAAGAAGCGAGAGTGACGG

TACCTGCAGAAAAAGCACCGGCTAACTACGTGCCAGCAGCCGCGGTAATA

CT

>543_HDA-1F_23 COL13-09TV

CGCATGAGCGAGCCTGACGGAGCATGCCGCGTGAAGGAAGAAGGCTCACGGGTCGTAAACTTCTTTTCTCGGAGAAGAATAAATGACGGTATCTGAGGAATAAGCATCGGCTAACTCTGTGCCAGCAGCCGCGGTAATACTCTGTGCCAGCAGCCGCGGTAATACA

>544_HDA-1F_24 COL13-09TV

GGGGACCTGATCAGCCATGCCGCGTGTGTGAAGAAGGCCTTATGGTTGTAAAGCACTTTAAGCGAGGAGGAGGCTCCTGTAGTTAAT

ACCTACAGAGAGTGGACGTTACTCGCAGAATAAGCACCGGCTAACTCTGT

GCCAGCAGCCGCGGTAATACA

>548_HDA-1F_28 COL13-09TV

TGTGAGGGAAAATGATGATGGTACCTC

ATAAGAAAGGACCGGCTAACTTCGTGCCAGCAGCCGCGGTAATACAATTA

>549_HDA-1F_29 COL13-09TV

ATGGGCGAGCCTGATCCAGCAATGCCGCGTGTGTGAAGAAGGCCTTCGGGTTGTAAAGCTCTTTTGTCCGGAAAGAAATCCTTGGCTCTAAT

ACAGTCGGGGGATGAC

>550_HDA-1F_30 COL13-09TV

GCGAGCCTGATCCAGCAATGCCGCGTGAGTGATGAAGGCCCTAGGGTTGTAAAGCTCTTTTACCCGGGAAGATAATGACTGTACCGGG

AGAATAAGCCCCGGCTAACTCCGTGCCAGCAGCCGCGGTAATACTGCAAC

G

>552_HDA-1F_32 COL13-09TV

GAGCGAGCCTGATCCAGCATGCCGCGTGCAGGATGAAGGCCTTCGGGTTGTAAACTGCTTTTGTACGGAACGAAAAAGCTTCTCCTAA

TACGAGAGGCCCATGACGGTACCGTAAGAATAAGCACCGGCTAACTACGT

GCCAGCAGCCGCGGTAATAC

>553_HDA-1F_33 COL13-09TV

GCGAGCCTGATCCAGCATGCCGCGTGCAGGATGAAGGCCCTCGGGTTGTAAACTGCTTTTGTACGGAACGAAAAGCCTGGGGCTAATA

TCCCCGGGTCATGACGGTACCGTAAGAATAAGCACCGGCTAACTACGTGC

CAGCAGCCGCGGTAATACA

>554_HDA-1F_34 COL13-09TV

TGTCACGAGAGTCTGACCCAGCCATGCCGCGTGAAGGATGAAGGTCCTCAGGATTGTAAACTTCTTTTATCTGGGACGAAAAAGGGAT

ACTCTTATCCACTTGACGGTACCAGAGGAATAAGCACCGGCTAACTCCGT

GCCAGCAGCCGCGGTAATAC

>555_HDA-1F_35 COL13-09TV

AGTCTGACGGAGCACGCCGCGTGAGCGAAGAAGGTCTTCGGATTGTAAAGCTCTGTTGTTAGGGAAGAACAAGTACCGTTC

GAATAGGGCGGTACTGTGACGGTACCTAACGAGAAAGCCACGGCTAACTA

CGTGCCAGCAGCCGCGGTAATACAA

>556_HDA-1F_36 COL13-09TV

AGCGCAGCCTGATCTAGCCATGCCGCGTGATTGATGAAGGCCTTAGGGTTGTAAAGATCTTTCAGCTGGGAAGATAATGACGGTACCAGCAGAAGAAGCCCCGGCTAACTCCGTGCCAGCAGCCGCGGTAATACTTCGTGCCAGCAGCCGCGGTAATACA

>557_HDA-1F_37 COL13-09TV

GCATGGAGCGCAGCCTGATGCAGCCATGCCGCGTGTATGAAGAAGGCCTTCGGGTTGTAAAGTACTTTCAGCGGGGAGGAAGGCGGTGGGGTTAATAGCTTCACCGATTGACGTTACCCGCAGAAGAAGCACCGGCTAACTCCGTGCCAGCAGCCGCGGTAATACACGCG

>558_HDA-1F_3 COL13-0AW5

CACCTGATCCAGCATGCCGCGTGTGTGAAGAAG

GCCTTCGGGTTGTAAAGCACTTTAGGTGGGGAAGAAAGGTAACGGGTGAA

TAATCTGTTATTTTGACGGTACCCGCAGAATAAGCACCGGCTAACTCTGT

GCCAGCAGCCGCGGTAATACA

>559_HDA-1F_4 COL13-0AW5

GAGAA

GCAAGGCCTTCGCGGTTGTACAGCACTTTAGGTGGGGAAGAAAGGTAACG

GGTGAATAATCTGTTATTTTGACGGTACCCGCAGAATAAGCACCGGCTAA

CTCTGTGCCAGCAGCCGCGGTAATACA

>560_HDA-1F_38 COL13-09TV

GGCACCTGATCCAGCAATGCCGCGTGTGTGAAGAAGGCCTTCGGGTTGTAAAGCACTTTAGGTGGGGAAGAAAGGTAACGGGTGAATAA

TCTGTTATTTTGACGGTACCCGCAGAATAAGCACCGGCTAACTCTGTGCC

AGCAGCCGCGGTAATACA

>561_HDA-1F_39 COL13-09TV

AGTCTGACGGAGCACGCCGCGTGAGTGATGAAGGTTTTCGGATCGTAAAACTCTGTTGTTAGGGAAGAACAAGTACCGGAGTAACTGCCGGTACCTTGACGGTACCTAACCAAAAAGCCACGGCTAACTACGTGCCAGCAGCCGCGGTAATACAACCTTCCCATGACGAA

>562_HDA-1F_40 COL13-09TV

AGCCTGATCCAGCAATGCCGCGTGCAGGATGAAGGCCTTCGGGTTGTAAACTGCTTTTGTACGGAACGAAAAAGCTTCTCCT

AATACGAGAGGCCCATGACGGTACCGTAAGAATAAGCACCGGCTAACTAC

GTGCCAGCAGCCGCGGTAATACA

>563_HDA-1F_41 COL13-09TV

GCGAGCCTGATCCAGCATGCCGCGTGCAGGATGAAGGCCCTCGGGTTGTAAACTGCTTTTGTACGGAACGAAAAGCCTGGGGCTAATAT

CCCCGGGTCATGACGGTACCGTAAGAATAAGCACCGGCTAACTACGTGCC

AGCAGCCGCGGTAATAC

>564_HDA-1F_43 COL13-09TV

CGCAGCCTGATCCAGCCATGCCGCGTGAGTGATGACGGCC

TTAGGGTTGTAAAGCTCTTTTCTCCGGGACGATAATGACGGTACCGGAGG

AATAAGCCCCGGCTAACTTCGTGCCAGCAGCCGCGGTAATACA

>565_HDA-1F_42 COL13-09TV

CGCGAAGCCTGACGGAGCACGCCGCGTGAGTGATGAAGGTCTTCGGATCGTAAAACTCTGTTATTAGGGAAGAACAAATGTGTAAGTA

ACTATGCACGTCTTGACGGTACCTAATCAGAAAGCCACGGCTAACTACGT

GCCAGCAGCCGCGGTAATAC

>566_HDA-1F_44 COL13-09TV

CAGCGAGCCTGATGCAGCACGCCGCGTGAGTGATGACGG

TCTTCGGATCGTAAAGCTCTGTCTTTGGGGACGATAATG

>567_HDA-1F_45 COL13-09TV

GAGCCTGATGCAGCACGCCGCGTGAGTGATGACGGT

CTTCGGATTGTAAAGCTCTGTCTTTGGGGACAATAATGA

>569_HDA-1F_47 COL13-09TV

AGGGACCTGATGCAGCAACGCCGCGTGAGTGATGACGGTCTTCGGATTGTAAAGCTCTGTCTTTGGGGACGATAATGACGGTACCCA

AGGAGGAAGCCACGGCTAACTACGTGCCAGCAGCCGCGGTAATACA

>570_HDA-1F_48 COL13-09TV

GGGTAGAATTCCAGGTGTAGCGGTGAAATGCGTAGAGATCTGGAGGA

ATACCGGTGGCCAAGGCGGCCCCCTGGACAAAGACTGACGCTCAGGTGCG

AAAGCGTGGGGAGCAAACAGGATTAGATACCCTGGTAGTCCACGCCGTAA

ACGATGTCTACTTGGAGGTTGTGCCCTTGAGGCGTGGCTTCCGGAGCTAA

CGCGTTAAGTCGACCGCCTGGGGAGTACGGCCGCAAGGTTAAAACTCAAA

TGAATTGACGGGGGCCCGCACAAGCGGTGGAGCATGTGGTTTAATTCTAT

GCACCGCGAAGAACCTTACCTACTCTTGACATCCAGAGAACTTTCCAGAG

ATGGATTGGTGCC

>571_HDA-1F_49 COL13-09TV

GAAGTCTGACGGAGCACGCCGCGTGAGCGATGAAG

GCCTTCGGGTCGTAAGCTCTGTTGTTAGGGAAGAACAAATAACGCAGTAA

CTGG

>572_HDA-1F_50 COL13-09TV

GCCTGATCCAGCAATGCCGCGTGAGTGATGA

AGGCCTTAGGGTTGTAAAGCTCTTTTACCCGGGATGATAATGACAGTACC

GGGAGAATAAGCTCCGGCTAACTCCGTGCCAGCAGCCGCGGTAATACAAG

AAC

>573_HDA-1F_51 COL13-09TV

GCGAGCCTGATCCAGCATGCCGCGTGAGTGATGAAGGCCT

TAGGGTTGTAAAGCTCTTTTACCCGGGATGATAATGACAGTACCGGGAGA

ATAAGCTCCGGCTAACTCCGTGCCAGCAGCCGCGGTAATACCTACGTGCC

AGCAGCCGCGGTAATACAAA

>574_HDA-1F_52 COL13-09TV

GGAAAGAAAAGCTTCCGGTTAATACCCGGGAGTCATGACGGTACCCAAAGAATAAGCACCGGCTAACTTCGTGCCAGCAGCCGCGGTAATACA

>575_HDA-1F_53 COL13-09TV

CGCAGCCTGATCCAGCAATGCCGCGTGTGTGAGAAGGCCTTCGGGTTGTAAAGCGCTTTTGTCGGGGAAGAAATCTTCCG

>576_HDA-1F_54 COL13-09TV

CACCCTGATCTAGCAATGCCGCGTGAGTGATGAAGGCCTTAGGGTTGTAAAGCTCTTTTACCTGGGAAGATAATGACGGTACCGGGAG

AATAAGCTCCGGCTAACTCCGTGCCAGCAGCCGCGGTAATACA

>578_HDA-1F_56 COL13-09TV

GCAGCTGATCCAGCAATGCCGCGTGAGTGATGAAGGCCTTAGGGTTGTAAAGCTCTTTTACCCGGGATGATAATGACAGTACCGG

GAGAATAAGCTCCGGCTAACTCCGTGCCAGCAGCCGCGGTAATACATCCT

GGCCGCCGccgcggtaattcaa

>579_HDA-1F_57 COL13-09TV

GGCGCAGCCTGATCCAGCCATGCCGCGTGTGTGAAGAAGGCCTTCGGGTTGTAAAGCACTTTTGTTGGGAAAGAAAAGCCTCCGGCTAATACCCGGGAGTCATGACGGTACCCAAAGAATAAGCACCGGCTAACTTCGTGCCAGCAGCCGCGGTAATACA

>580_HDA-1F_58 COL13-09TV

GGGCACCCTGATCTAGCCATGCCGCGTGAGTGATGAAGGCCTTAGGGTTGTAAAGCTCTTTCAGCTGGGAAGATAATGACGGTACCAGC

AGAAGAAGCCCCGGCTAACTCCGTGCCAGCAGCCGCGGTAATACAGAGAT

GC

>581_HDA-1F_59 COL13-09TV

GCAATGCCGCGTGAGTGATAGAAG

GCCTTTTGGTTGTAAAGCTCTTTTACCGAGGAAGAAATGCCGAGA

>582_HDA-1F_60 COL13-09TV

GGCGCAGCCTGATCCAGCCATGCCGCGTGTGTGATAGAAGGCCTTCGGGTTGTAAAGCTCTTTTGTTGGGAAAGAAAATGAC

>584_HDA-1F_62 COL13-09TV

GGCGCAGCCTGATCCAGCCATGCCGCGTGAGTGATGAAGGCCTTAGGGTTGTAAAGCTCTTTTATCCGGGACGATAATGACGGTACCGGAG

GAATAAGCCCCGGCTAACTTCGTGCCAGCAGCCGCGGTAATACATCACCG

CACCGCGGatac

>585_HDA-1F_63 COL13-09TV

GGACGAGTCTGACCGAGCACGCCGCGTGAGTGAAGAAGGTTTTCGGATCGTAAAACTCTGTTGTTAGAGAAGAACGTTGTGTAGAGTGGAAAATTACACAAGTGACGGTATCTAACCAGAAAGGGACGGCTAACTACGTGCCAGCAGCCGCGGTAATACA

>586_HDA-1F_64 COL13-09TV

GCGAAGCCTGACGGAGCATGCCGCGTGAAGGAAGAAGGCTCACGGGTCGTAAACTTCTTTTCTCGGAGAAGAATAAATGACGGTATCT

GAGGAATAAGCATCGGCTAACTCTGTGCCAGCAGCCGCGGTAATACAAAT

TC

>587_HDA-1F_65 COL13-09TV

GCAGTCTGATCCAGCCATGCCGCGTGTGTGAGAAG

GCCTTCGGGTTGTAAAGCACTTTTGTTGGGAAAGAAAAGCCTGCGGCTA

>588_HDA-1F_66 COL13-09TV

GAGCCTGATGCAGCATGCCGCGTGAGTGATGAAGGCC

TTAGGGTTGTAAACCTCTTTTACCCGGGAAGATAATGACAGTAC

>589_HDA-1F_67 COL13-09TV

AGCCTGACGCGAGCCTGCCGCGTGGGGGTAGAAGGCCCATCGGGTCGTGAACTTCTTTTCCCGGAGAAGAAGCAATGACG

GTATCTGGGGAATAAGCATCGGCTAACTCTGTGCCAGCAGCCGCGGTAAT

ACCGTGCCAGCAGCCGCGGTAATACA

>590_HDA-1F_68 COL13-09TV

CGAGCCTGACGGAGCATGCCGCGTGGAGGTAGAAGGCCCACGGGTCGTGAACTTCTTTTCCCGGAGAAGAAGCAATGACGGTATCT

GGGGAATAAGCATCGGCTAACTCTGTGCCAGCAGCCGCGGTAATACACGT

>591_HDA-1F_69 COL13-09TV

ATGAGCGAGCCTGACGGAGCACGCCGCGTGAGTGATGAAGGTCTTCGGATCGTAAAACTCTGTTATTAGGGAAGAACAAATGTGTAAGTAACTATGCACGTCTTGACGGTACCTAATCAGAAAGCCACGGCTAACTACGTGCCAGCAGCCGCGGTAATAC

>592_HDA-1F_70 COL13-09TV

AATGACGGTACTTGAGGAGG

AAGCCCCGGCTAACTACGTGCCAGCAGCCGCGGTAATACA

>593_HDA-1F_71 COL13-09TV

GCGAGCCTGATGCAGCAACGCCGCGTGAGTGATGACGGCTCTTCGGGTTGTAAAGCTCTGTCTTCGGGGACGATAATGAC

>594_HDA-1F_72 COL13-09TV

AGCCTGATGCAGCCCGCCGCGTGAGTGATGAAGGCC

TTCGGGTTGTAAAGCTCTGTCCTTGGGGCGAT

>595_HDA-1F_73 COL13-09TV

AGCCTGATGCAGCCCGCCGCGTGAGTGATGAA

GGCCTTCGGGTTGTAAAGCTCTTTTGTTGGGAAGAA

>596_HDA-1F_74 COL13-09TV

CGAGTCTGATCCAGCACGCCGCGTGAGTGATGAAGGTCTTCGGATCTGTAAAGCTCTGTTGTCAGAGAAGAACGAGTGTGAG

AGTGGAAAGTTCACACTGGGACGG

>597_HDA-1F_75 COL13-09TV

GGCGCAGCCTGATCCAGCCATGCCGCGTGTGTGAAGAAGGCCTTCGGGTTGTAAAGCACTTTTGTTGGGAAAGAAAAGCGCGGAGTTAATACCCCCGCGTCATGACGGTACCCAAAGAATAAGCACCGGCTAACTTCGTGCCAGCAGCCGCGGTAATAC

>600_HDA-1F_78 COL13-09TV

CAGCCTGATGCAGCCCGCCGCGTGAGTGATGAAG

GCCTTCGGGTTGTAAAGCTCTGTCTTTGGGGACGATAT

>601_HDA-1F_79 COL13-09TV

GCGCGAGCCTGACGGAGCACGCCGCGTGAGTGATGAAGGTCTTCGGATCGTAAAACTCTGTTATTAGGGAAGAACAAATGTGTAAGTAACTATGCACGTATTGACGGTACCTAATCAGAAAGCCACGGCTAACTACGTGCCAGCAGCCGCGGTAATACA

>603_HDA-1F_81 COL13-09TV

CAGCCTGATGCAGCATGCCGCGTGAGTGATGAAG

GCCTTCGGGTTGTAAACTGCTTTTGTCGGGACG

>604_HDA-1F_82 COL13-09TV

GGAGCCTGATGCAGCACGCCGCGTGAGTGATGACGGCCTTCGGATTGTAAAGCTCTGTCTTTGGGGACGATAATGACGGTACCCAAG

GAGGAAGCCACGGCTAACTACGTGCCAGCAGCCGCGGTAATACAATACAC

C

>605_HDA-1F_83 COL13-09TV

GCGAGCCTGATGCAGCACGCCGCGTGAGTGATGAAGGTCTTCGGATCGTAAAACTCTGTTATTAGGGAAGAACAAACGTGTAAGTAACTGTGCACGTCTTGACGGTACTTAATCAGAAAGCCACGGCTAACTACG

TGCCAGCAGCCGCGGTAATAC

>606_HDA-1F_84 COL13-09TV

CGCAGCCTGATGAGCACGCCGCGTGAGTGATGAAGGTCTTCGGATCGTAAAACTCTGTTATTAGGGAAGAACAAACGTGTAAGTAACTGTGCACGTCTTGACGGTACCTAATCAGAAAGCCACGGCTAACTACGTGCCAGCAGCCGCGGTAATACAACA

>607_HDA-1F_85 COL13-09TV

AGCGAGCCTGATGGAGCACGCCGCGTGAGTGATGAAGGTCTTCGGATCGTAAAACTCTGTTATTAGGGAAGAACAAATGTGTAATTAACTATGCACGTCTTGACGGTACCTAATCAGAAAGCCACGGCTAACAACGTGCCAGCAGCCGCGGTAATACAACA

>609_HDA-1F_87 COL13-09TV

CGCAGCCTGATGCAGCACGCCGCGTGAGTGATGAAG

GCCTTCGGATTGTAAAGCTCTGTCTTTGGGGACA

>610_HDA-1F_88 COL13-09TV

GAGCGAGCCTGACGGAGCACGCCGCGTGAGTGATGAAGGTCTTCGGATCGTAAAACTCTGTTATTAGGGAAGAACAAATGTGTAAG

TAACTATGCACGTCTTGACGGTACCTAATCAGAAAGCCACGGCTAACTAC

GTGCCAGCAGCCGCGGTAATAC

>611_HDA-1F_89 COL13-09TV

GCCTGATGCAGCAATGCCGCGTGAGTGATGAAGGCCTTCGGGTTGTAAAGCTCTTTTGTTGGGAAAGATA

>612_HDA-1F_90 COL13-09TV

AAGGATGATAATGACGGTACTTGAAG

AGGAAGCCCCGGCTAACTACGTGCCAGCAGCCGCGGTAATACACGT

>613_HDA-1F_5 COL13-0AW5

GCAGCCTGATCCAGCCATCCCGCGTGAAGGAT

TAACGTCCTATGGATTGTAAACTTCTTTTATACAGGGATAAACCTACTTA

CGTGTAAGTAGCTGAAGGTACTGTATGAATAAGCACCGGCTAACTCCGTG

CCAGCAGCCGCGGTAATACAAATA

>614_HDA-1F_92 COL13-09TV

GGATGCGTGCAGCCTGACCAGCCATCCCGCGTGAAGGACGACTGCCCTATGGGTTGTAAACTTCTTTTGTATAGGGATAAACCTACCCTCGT

GAGGGTAGCTGAAGGTACTATACGAATAAGCACCGGCTAACTCCGTGCCA

GCAGCCGCGGTAATAC

>615_HDA-1F_93 COL13-09TV

ATGGGCGCAGCCTGATCCAGCCATGCCGCGTGTGTGAAGAAGGCCTTCGGGTTGTAAAGCACTTTTGTTGGGAAAGAAAAGCGCGGAGTTAATACCCCCGCGTCATGACGGTACCCAAAGAATAAGCACCGGCTAACTTCGTGCCAGCAGCCGCGGTAATAC

>618_HDA-1F_7 COL13-0AW5

GCGCAGCCTGATCAGCCATGCCGCGTGTGTGAAGAA

GGCCTTCGGGTTGTAAAGCACTTTTGTTGGGAAAGAAAAGCCCGTGGTT

>620_HDA-1F_95 COL13-09TV

TCAGCGCAGCCTGATCCAGCCATGCCGCGTGTGTGATAGAAGGCCTTCGGGTTGTAAAGCTCTTTTGTTGGGGAAGAAAAGCACTT

GGTTAATACCC

>621_HDA-1F_96 COL13-09TV

AGCGCAGCCTGATCCAGCCATGCCGCGTGTGTGAAGAAGGCCTTCGGGTTGTAAAGCTCTTTTGTTGGGAAAGAAAAGCTTCCGGCT

AATA

>622_HDA-1F_91 COL13-09TV

TGCAGCGCAGCCTGATCCAGCCATGCCGCGTGAGTGATGAAGGCCT

TCGGGTTGTAAAGCTCTTTTGTTGGGGAAGAAAATGACC

>623_HDA-1F_67 COL13-09S0

CAGCGCAAGCCTGATCCAGCCATGCCGCGTGTGTGAAGA

AGGCCTTCGGGTTGTAAAGCACTTTTGTTGGGAAAGAAAAGCCTCCGGCT

AATACCCGGGAGTCATGACGGTACCCAAAGAATAAGCACCGGCTAACTTC

GTGCCAGCAGCCGCGGTAATACA

>624_HDA-1F_68 COL13-09S0

AGCGCAGCCTGATCCAGCCATGCCGCGTGTGTGAAGAAGGC

CTTCGGGTTGTAAAGCACTTTTGTTGGGAAAGAAAAGCGCGGAGTTAATA

CCCCCGCGTCATGACGGTACCCAAAGAATAAGCACCGGCTAACTTCGTGC

CAGCAGCCGCGGTAATAC

>625_HDA-1F_9 COL13-0AW5

ATGGGGGCACCCTGACCCAGCGACGCCGCGTGAGGGAAGACA

GCCTTCGGGTTGTAAACCTCTGTTGCAGGGGAAGAAGGACGTGACGGTAC

CCTGCGAGGAAGCTCCGGCTAACTACGTGCCAGCAGCCGCGGTAATACAA

C

>626_HDA-1F_10 COL13-0AW5

CATGGGGGCACCCTGACCCAGCGACGCCGCGTGAGGGAAGACA

GCCTTCGGGTTGTAAACCTCTGTTGCAGGGGAAGAAGGACGTGACGGTAC

CCTGCGAGGAAGCTCCGGCTAACTACGTGCCAGCAGCCGCGGTAATACT

>627_HDA-1F_11 COL13-0AW5

AGCGAGCCTGACCCAGCACGCCGCGTGAAGGATGAAG

TATTTCGGTATGTAAACTTCGAAAGAATGGGAAGAATAAATGACGGTACC

ATTTATAAGCTCCGGCTAACTACGTGCCAGCAGCCGCGGTAATACAGCCG

TGCCCCCCGCCGCGA

>628_HDA-1F_12 COL13-0AW5

GGCGCAGCCTGATCAGCATGCCGCGTGAGTGATGAAGGC

CTTCGGGTTGTAAACTTCTTTTAGTGGG

>629_HDA-1F_13 COL13-0AW5

TATGGGCGAGCCTGATCCAGCCATGCCGCGTGCAGGATGAAGGCCT

TCGGGTTGTAAACTGCTTTTGTACGGAACGAAAAGGTCTTTTCTAATACA

GAAGGCTCATGACGGTACCGTAAGAATAAGCACCGGCTAACTACGTGCCA

GCAGCCGCGGTAATAC

>630_HDA-1F_14 COL13-0AW5

TATGGGTGCAGCCTGATCCAGCCATCCCGCGTGAAGGACGACT

GCCCTATGGGTTGTAAACTTCTTTTGTATAGGGATAAACCTACCCTCGTG

AGGGTAGCTGAAGGTACTATACCAATAAACACCGGCTAACTCCGTGCCAG

CAGCCGCGGTAATACAATCA

>632_HDA-1F_16 sCOL13-0AW5

GCGCAGCCTGATCAGCATGCCGCGTGTGTGAAGAAGG

CCTTCGGGTTGTAAAGCGCTTTTGTTGGGGATGAAATGTGCTGGG

>633_HDA-1F_17 COL13-0AW5

GCGCAGCCTGATCAGCATCGCCGCGTGTGTGAAGAAGG

CCTTCGGGTTGTAAAGTGCTTTTGTTGGGGAAGAAATCTTCTG

>634_HDA-1F_18 COL13-0AW5

GCGAGCCTGATGCAGCAATGCCGCGTGATGATGAAGGC

CTTCGGGTGTAACTTC

>635_HDA-1F_19 COL13-0AW5

TGCAGCCACGCCGCGTGAGTGATAGAA

GGCCTTAGGATTTGTAACT

>636_HDA-1F_20 COL13-0AW5

CATGAGCGAGCCTGACGCAGCCATGCCGCGTGAATGATGAAG

GTCTTAGGATTGTAAAATTCTTTCACCGGGGACGATAATGACGGTACCCG

GAGAAGAAGCCCCGGCTAACTTCGTGCCAGCAGCCGCGGTAATACTAAGT

GCCAGCAGCCGCGGTAATACA

>637_HDA-1F_21 COL13-0AW5

GAGTATGGGCGCAGCCTGATCCAGCATGCCGCGTGAGTGATGAAGGCC

TTAGGGTTGTAAAGCTCTTTTACCAGGGATGATAATGACAGTACCTGGAG

AATAAGCTCCGGCTAACTCCGTGCCAGCAGCCGCGGTAATACTAAGTGGC

AGCA

>638_HDA-1F_22 COL13-0AW5

GCGCAGCCTGATCCAGCATGCCGCGTGAGTGATGAAGGCC

TTAGGGTTGTAAAGCTCTTTTACCAGGGATGATAATGACAGTACCTGGAG

AATAAGCTCCGGCTAACTCCGTGCCAGCAGCCGCGGTAATACAACC

>639_HDA-1F_23 COL13-0AW5

CATGGGCGCAGCCTGATCCAGCAATGCCGCGTGAGTGATGAA

GGCCTTAGGGTTGTAAAGCTCTTTTACCAGGGATGATAATGACAGTACCT

GGAGAATAAGCTCCGGCTAACTCCGTGCCAGCAGCCGCGGTAATACAA

>640_HDA-1F_24 COL13-0AW5

GCGAAGCCTGATCCAGCAATGCCGCGTGCAGGATGAAG

GCCCTCGGGTTGTAAACTGCTTTTGTACGGAACGAAAAGCCTGGGGCTAA

TATCCCCGGGTCATGACGGTACCGTAAGAATAAGCACCGGCTAACTACGT

GCCAGCAGCCGCGGTAATACCA

>641_HDA-1F_25 COL13-0AW5

GCATGGAGCGCAGCCTGATGCAGCCATGCCGCGTGTATGAAGAA

GGCCTTCGGGTTGTAAAGTACTTTCAGCGGGGAGGAAGGCGATGAGGTTA

ATAACCGCATCGATTGACGTTACCCGCAGAAGAAGCACCGGCTAACTCCG

TGCCAGCAGCCGCGGTAATACA

>642_HDA-1F_26 COL13-0AW5

CGCAGTCTGATGCAGCGACGCCGCGTGAGTGGATGACG

GCTCTTCGGGTTGTAAACCTCTTTCGTCAGGGAAGAACCGATGGTGACTG

AACCTG

>643_HDA-1F_27 COL13-0AW5

CGCAGCCTGATCCAGCCATGCCGCGTGAGTGATGAA

GGCCTTAGGGTTGTAAAGCTCTTTCACCGGAGAAGATAATGACGGTATCC

GGAGAAGAAGCCCCGGCTAACTTCGTGCCAGCAGCCGCGGTAATACAATA

CA

>644_HDA-1F_28 COL13-0AW5

CAGCATGGGCGCAGCCTGATGCAGCACGCCGCGTGAGGGATGACGGCCT

TCGGGTTGTAAACCTCTTTTAGCAGGGAAGAAGCGAGAGTGACGGTACCT

GCAGAAAAAGCACCGGCTAACTACGTGCCAGCAGCCGCGGTAATACA

>645_HDA-1F_29 COL13-0AW5

ATGGACGAAGTCTGACGGAGCACGCCGCGTGAGCGAAGAAG

GTCTTCGGATTGTAAAGCTCTGTTGTTAGGGAAGAACAAGTACCGTTCGA

ATAGGGCGGTACCGTGACGGTACCTAACGAGAAAGCCACGGCTAACTACG

TGCCAGCAGCCGCGGTAATAC

>646_HDA-1F_30 COL13-0AW5

GGGGACCCTGATGCAGCGACGCCGCGTGAGTGATGAAG

CCCTTCGGGGTGTAAAGCTCTTTCGGCCCGGACGATAATGACGGTACGGG

AAGAAGAAGCTGCGGCTAACTACGTGCCAGCAGCCGCGGTAATACT

>647_HDA-1F_31 COL13-0AW5

ACGCAGTCTGACCGAGCACGCCGCGTGAGTGATGAAG

GTTTTCGGATCGTAAAGCTCTGTTGTAAGAGAAGAACGAGTGTGAGAGTG

GAAAGTTCACACTGTGACGGTATCTTAC

>649_HDA-1F_1 COL13-0B80

CATGGGCGCAGCCTGATCCAGCCATGCCGCGTGAGTGATGAAG

GCCCTAGGGTTGTAAAGCTCTTTTGTGCGGGAAGATAATGACGGTACCGC

AAGAATAAGCCCCGGCTAACTTCGTGCCAGCAGCCGCGGTAATACTGAGC

AGT

>650_HDA-1F_2 COL13-0B80

ATGGGCGCAGCCTGATCCAGCCATGCCGCGTGAGTGATGAA

GGCCCTAGGGTTGTAAAGCTCTTTTGTGCGGGAAGATAATGACGGTACCG

CAAGAATAAGCCCCGGCTAACTTCGTGCCAGCAGCCGCGGTAATACTGCT

C

>651_HDA-1F_3 COL13-0B80

CATGGGGGCACCCTGATCCAGCCATGCCGCGTGTGTGAAGAAG

GCCTTCGGGTTGTAAAGCACTTTCATTGGTGAGGAAGGATATAAAATTAA

TACTTTTATGTATTGACGTTAACCAAAGAAGAAGCACCGGCTAACTCCGT

GCCAGCAGCCGCGGTAATAATGA

>652_HDA-1F_4 COL13-0B80

GGCACCCTGATCCAGCCATGCCGCGTGTGTGAAGAA

GGCCTTCGGGTTGTAAAGCACTTTCATTGGTGAGGAAGGATATAAAATTA

ATACTTTTATGTATTGACGTTAACCAAAAAAAAAGCACCGGCTAACTCCG

TGCCAGCAGCCGCGGTAAT

>653_HDA-1F_5 COL13-0B80

TGAGGGACCCTGACGCAGCACGCCGCGTGAGTGATGAAG

GCCTTCGGGTTGTAAAGCTCTTTCGGTTGGGAAGAAGGGGTATTTGGTTA

ATAAGCAAGTACTTTGATGGTACCAAAAGAAGAAGCACCGGCAAACTTCG

TGCCAGCAGCCGCGGTAATA

>654_HDA-1F_6 COL13-0B80

GCAGCGAAGCCTGATCCAGCCATGCCGCGTGCGGGATGAAG

GCCTTCGGGTTGTAAACTGCTTTTGTACGGAACGAAAAGGTTTGGCCTAA

TAAGCTGAGCTCATGACGGTACCGTAAGAATAAGCACCGGCTAACAACGT

GCCAGCAGCCGCGGTAATACAA

>655_HDA-1F_7 COL13-0B80

GGCGAGCCTGATCCAGCATGCCGCGTGAGTGATGAAGGC

CTTAGGGTTGTAAAGCTCTTTTACCCGGGATGATAATGACAGTACCGGGA

GAATAAGCTCCGGCTAACTCCGTGCCAGCAGCCGCGGTAATACCTACGTG

CCAGCAGCCGCGGTAATACA

>656_HDA-1F_8 COL13-0B80

CGCATGCGCGAGCCTGACGGAGCACGCCGCGTGAGTGATGAAG

GTCTTCGGATCGTAAAACTCTGTTATTAGGGAAGAACAAATGTGTAAGTA

ACTATGCTCGTCTTGACGGTACCTAGTCACAAAGCCACG

>657_HDA-1F_9 COL13-0B80

CGCATGAGCGAGCCTGACGGAGCACGCCGCGTGAGTGATGAAG

GTCTTCGGATCGTAAAACTCTGTTATTAGGGAAGAACAAATGTGTAAGTA

ACTATGCACGTCTTGACGGTACCTAATCAGAAAGCCACGGCTAACTACGT

GCCAGCAGCCGCGGTAATACA

>658_HDA-1F_10 COL13-0B80

GGGCGAGCCTGACGGAGCATGCCGCGTGAAGGAAGAAGGCTC

ACGGGTCGTAAACTTCTTTTCTCGGAGAAGAATAAATGACGGTATCTGAG

GAATAAGCATCGGCTAACTCTGTGCCAGCAGCCGCGGTAATACAG

>659_HDA-1F_11 COL13-0B80

AGCGAGCCTGACGGAGCAACGCCGCGTGAGTGATGAAGG

TCTTCGGATCTGTAAAACTCTGTTATTAGGGAAGAACAAATGTGTAAGTA

ACTATGCACGTCTTGACGGTACCTAATCAGAAAGCCACGGCTAACTACGT

GCCAGCAGCCGCGGTAATACA

>660_HDA-1F_12 COL13-0B80

CGGGTTGTAAACTGCTTTTGTACGGAACGAAAAAGCTTCTCCTA

ATACGAGAGGCCCATGACGGTACCGTAAGAATAAGCACCGGCTAACTACG

TGCCAGCAGCCGCGGTAATACA

>661_HDA-1F_13 COL13-0B80

GCATGGGCGAGCCTGATCCAGCATGCCGCGTGCAGGATGAAGG

CCTTCGGGTTGTAAACTGCTTTTGTACGGAACGAAAAAGCTTCTCCTAAT

ACGAGAGGCCCATGACGGTACCGTAAGAATAAGCACCGGCTAACTACGTG

CCAGCAGCCGCGGTAATACA
